# Liquid chromatography–high-resolution tandem mass spectrometry of anatoxins, including new conjugates and reduction products

**DOI:** 10.1007/s00216-023-04836-y

**Published:** 2023-07-29

**Authors:** Daniel G. Beach, Lydia Zamlynny, Melanie MacArthur, Christopher O. Miles

**Affiliations:** Biotoxin Metrology, National Research Council Canada, 1411 Oxford St., Halifax, NS Canada

**Keywords:** Cyanobacteria, Cyanotoxin, Anatoxin-a, Non-target analysis, High-resolution mass spectrometry, LC–HRMS

## Abstract

Anatoxins (ATXs) are a potent class of cyanobacterial neurotoxins for which only a handful of structural analogues have been well characterized. Here, we report the development of an LC–HRMS/MS method for the comprehensive detection of ATXs. Application of this method to samples of benthic cyanobacterial mats and laboratory cultures showed detection of several new ATXs. Many of these result from nucleophilic addition to the olefinic bond of the α,β-unsaturated ketone functional group of anatoxin-a (ATX) and homoanatoxin-a (hATX), analogous to the conjugation chemistry of microcystins, which contain similar α,β-unsaturated amide functionality. Conjugates with glutathione, γ-glutamylcysteine, methanethiol, ammonia, methanol and water were detected, as well as putative C-10 alcohol derivatives. Structural confirmation was obtained by simple and selective analytical-scale semisynthetic reactions starting from available ATX standards. Methanol, water and ammonia conjugates were found to result primarily from sample preparation. Reduction products were found to result from enzymatic reactions occurring primarily after cell lysis in laboratory cultures of *Kamptonema formosum* and *Cuspidothrix issatschenkoi*. The relative contributions of the identified analogues to the anatoxin profiles in a set of 22 benthic-cyanobacterial-mat field samples were estimated, showing conjugates to account for up to 15% of total ATX peak area and 10-hydroxyanatoxins up to 38%. The developed methodology, new analogues and insight into the chemical and enzymatic reactivity of ATXs will enable a more comprehensive study of the class than possible previously.

**Supplementary information:**

The online version contains supplementary material available at 10.1007/s00216-023-04836-y.

## Introduction

Anatoxins are a class of potent neurotoxins produced by cyanobacteria that are increasingly being recognized as a threat to drinking and recreational water supplies worldwide [1, 2]. Anatoxin-a (ATX) was first discovered in cyanobacteria isolated from a lake in western Canada in the 1970s [3]. Anatoxins are produced by a range of globally distributed cyanobacterial taxa including the genera *Dolichospermum*, *Microcoleus*, *Tychonema*, *Cuspidothrix* and *Kamptonema*. Toxicologically, ATX is an agonist of nicotinic acetylcholine receptors, ultimately leading to respiratory arrest in exposed animals [4]. Intoxication of livestock [4–6], pet dogs [7–13] and wildlife [11, 14] by anatoxins has been relatively widely reported across multiple continents. Recently, a case of human poisoning from ATX was documented in France, though the source of the toxin, which was consumed via edible marine invertebrates, remains unknown [15]. The production of ATXs by marine and brackish benthic cyanobacteria has rarely been reported [12, 16], but could explain this incident.

**Fig. 1:**
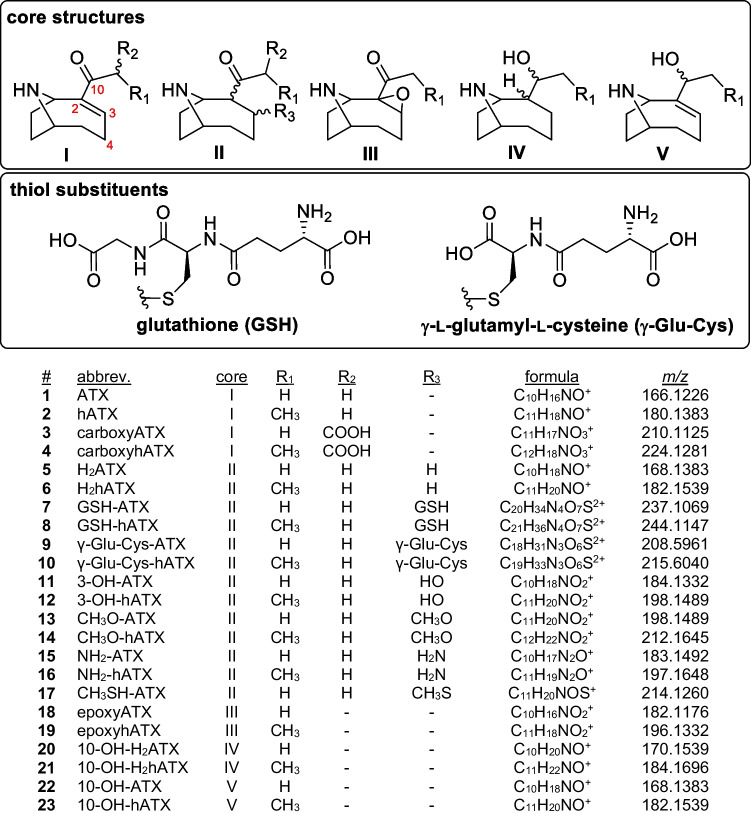
Structures, abbreviations and exact *m*/*z* ([M + H]^+^ or [M + 2H].^2+^, as indicated) of ATXs detected in field and culture samples in this study. An expanded version including isomer label assignments and retention time information is provided in the Supplementary information (Fig. S1)

Comprehensive analysis of cyanotoxins is challenging because they belong to distinct chemical classes: microcystins (MCs), ATXs, cylindrospermopsins, saxitoxins, each with differing chemical structures, chromatographic and mass spectral properties and modes of toxicity. Additionally, a potentially large number of structural variants exist within each class. This is especially true for saxitoxins [17] and MCs [18], for which over 50 and 300 analogues have been reported, respectively. On the other hand, only a relatively small number of cylindrospermopsins and ATXs have been reported. Structural variants of ATX include the other parent compound homoanatoxin-a (hATX) [19] and dihydro- [20–22], epoxy- [22, 23] and carboxy- [20, 24] analogues of ATX and hATX, as well as rarely reported 4-hydroxy- and 4-keto- analogues [25–27] (Fig. 1). Dihydroanatoxin-a (H_2_ATX) was originally thought to be a degradation product of ATX [22, 28] exhibiting lower toxicity [29], but has since been shown, along with dihydrohomoanatoxin-a (H_2_hATX), to be a biosynthetic product of cyanobacteria [30] and to exhibit similar toxicity to ATX in mouse feeding studies [21]. In addition to these naturally occurring analogues, a wide range of modified ATXs, including C10-hydroxylated anatoxin-a (originally termed anatoxinol), have been synthesized in the study of structure–activity relationships with respect to nicotinic acetylcholine receptors [31, 32]. These studies have demonstrated the presence and conformation of the N-9 amine hydrogen bond donor and C-10 ketone hydrogen bond acceptor to be critical factors for ATX toxicity [32], and hydrogen bonding interactions between these functional groups of ATX and sites within the acetylcholine-binding protein have recently been confirmed by X-ray crystallography [33].

An additional confounding factor in cyanotoxin analysis is the potential for the formation of bound forms of toxins. Binding of MCs in tissues of exposed animals can occur through a specific interaction with protein phosphatase enzymes, the molecular target for MC toxicity, the active site of which includes a nearby thiol-containing cysteine (Cys) residue that binds covalently with the α,β-unsaturated amide of the *N*-methyldehydroalanine (Mdha^7^) moiety found at position 7 in most MCs [34]. This same chemistry also leads to non-specific covalent binding of MCs to other thiol-containing biomolecules such as glutathione (GSH) and its degradation products (γ-Glu-Cys, Cys-Gly and Cys), yielding small-molecule conjugates that can be detected using liquid chromatography–mass spectrometry (LC–MS) methods [35–37]. This chemistry has been useful for the analytical detection [38] and structural elucidation [39] of new MCs and has been shown to be reversible [40]. It is also considered to be the main contributing factor for poor recoveries of MCs from exposed tissues and the commonly observed disagreement between targeted LC–MS analysis and techniques more suited for measuring total MCs (e.g. ELISA or MMPB oxidation) [41, 42]. Despite the fact that ATX and hATX both possess analogous α,β-unsaturated ketones, similar products resulting from nucleophilic attack of thiols at this position in ATX have not, to the best of our knowledge, been reported.

Anatoxin analysis by LC–MS in general is challenging due to the high background at low *m*/*z* in mass spectrometry and the polarity of ATX, making it is relatively poorly retained in both reverse-phase and hydrophilic-interaction liquid chromatography [43, 44]. Additionally, the stability of ATX is generally considered to be poor under typical environmental conditions due to its photochemical reactivity and poor stability at high pH, as often observed in late-stage cyanobacterial blooms [45]. Laboratory experiments have shown a half-life on the order of 1–2 h under UV irradiation and 4–10 days without irradiation at pH 9. In both cases, mouse bioassay measurements of total toxicity showed a decrease in toxicity corresponding to loss of ATX [45]. Reported degradation products of ATXs include a variety of uncharacterized isomeric products of photochemical rearrangements [45], oxidation to epoxides [22, 23], hydration [46] and methanol addition [26]. However, in many cases, these products have only been tentatively identified.

Here, we report the development of an LC–high-resolution tandem mass spectrometry (LC–HRMS/MS) method for the comprehensive detection of ATXs. This method was used to detect several new ATXs in benthic-cyanobacterial-mat and cyanobacterial culture samples. We then applied semisynthetic strategies from available standards for structural confirmation. Finally, we screened a set of cyanobacterial-mat field samples from the highly impacted Wolastoq (Saint John River) in New Brunswick, Canada, to help determine the potential relevance of these ATX derivatives to research and regulatory monitoring of ATXs.

## Experimental

### Chemicals and reagents

A certified reference material calibration solution for ( +)-anatoxin-a (NRC CRM-ATX-a [47], 4.96 ± 0.18 µg/mL) in 9:91 v/v CH_3_OH–H_2_O with 0.01% acetic acid and a pilot-scale freeze-dried cyanobacterial dietary supplement matrix reference material characterized for multiple classes of cyanotoxins (RM-BGA [43]) were from the National Research Council of Canada (Halifax, NS, Canada). Optima LC–MS-grade methanol and acetonitrile were from Fisher (Ottawa, ON, Canada). Glutathione (GSH), γ-l-glutamyl-l-cysteine, l-cysteine, sodium thiomethoxide, l-cysteinylglycine, formic acid (LC–MS grade), ammonium bicarbonate, sodium borohydride, sodium cyanoborohydride and sodium bicarbonate were from Sigma-Aldrich (Oakville, ON, Canada). Deionized H_2_O was produced by passing distilled H_2_O through a Milli Q Reference A + System (Millipore, Bedford, MA, USA). Standards for hATX and H_2_ATX (10 µg/mL in 3:1 v/v CH_3_OH–H_2_O with 0.1% acetic acid) were from Eurofins Abraxis (Warminster, PA, USA).

### Samples and sample preparation

Benthic-cyanobacterial-mat field samples were collected from the Wolastoq (Saint John River) near Fredericton, NB, Canada, in the summer of 2019. Sub-samples (1 g) of homogenized mats were vortex-mixed with 1 mL of CH_3_OH containing 0.1% formic acid and then centrifuged at 21,000 g for 10 min. RM-BGA was extracted with 3:1 v/v CH_3_OH–H_2_O containing 0.1% acetic acid (1 mL/mg). All samples were filtered to 0.45 µm using Durapore PVDF membrane centrifugal filters (Millipore, Bedford, MA, USA) prior to analysis.

Clonal cultures of *Kamptonema formosum* strain NIVA-CYA 92, from the Norwegian Culture Collection of Algae, and *Cuspidothrix issatschenkoi* strain CAWBG02, from the Cawthron Institute’s Culture Collection of Microalgae, were grown in Z8 and BG11 freshwater media, respectively, at 18 °C under a 14:10-h light:dark cycle in a model E7/2 dual-compartment plant growth chamber (CONVIRON, Winnipeg, MB, Canada). Sub-samples (15 mL) of late-stage cultures were centrifuged (8000 g), the supernatant decanted and the concentrated sample subjected to freeze–thaw cell lysis and extracted with CH_3_OH, as described above for benthic samples.

### Semisynthesis of ATX derivatives

Thiol conjugates of ATX and hATX were prepared by dissolving 1 mg of each thiol in 500 µL of CH_3_OH (100 µL for Cys) and then mixing with 500 µL of 100 mM NaHCO_3_ (1 M for Cys reaction). Each reaction mixture was vortex-mixed for 1 min, and an ATX or hATX standard added to a final concentration of 84 ng/mL ATX or 145 ng/mL hATX, respectively, vortex mixed for 1 min, and left overnight at ambient temperature to react.

Anatoxin-a and hATX standards were reduced by diluting them in 1 mL of 1:1 v/v CH_3_OH–H_2_O (to 84 ng/mL ATX and 167 ng/mL hATX), vortex-mixing with 1 mg of NaBH_4_ and leaving the reaction to stand at ambient temperature overnight. Anatoxin, hATX and H_2_ATX (38 ng/mL) standards were also reduced with NaBH_3_CN, as for NaBH_4_, but in the presence of 0.1% formic acid.

### LC–HRMS methods

LC–HRMS analyses were performed using an Agilent 1290 Infinity II liquid chromatography system (Agilent, Santa Clara, CA, USA) coupled to a Q Exactive HF Orbitrap mass spectrometer with a HESI-II electrospray ionization source (Thermo Fisher Scientific, Waltham, MA, USA). The LC method used an Acquity 1.8 μm HSS T3 column (150 $\times$ 2.1 mm; Waters, Milford, MA, USA) held at 40 °C and mobile phases A and B of 0.1% (v/v) formic acid in H_2_O and acetonitrile, respectively. A 0.2 mL/min linear elution gradient started at 2% B and increased to 11% B at 25 min. At 25.1 min, the concentration of mobile phase B was increased to 95% and held until 30 min. This was followed by a 10-min re-equilibration with 2% B. An injection volume of 1 µL was used in most cases with additional injections of 0.1 µL used to improve retention time alignment of high-level samples. Sheath and auxiliary gas flow rates were 35 and 10 units, respectively. The capillary and heater temperatures were set to 350 °C and 300 °C, respectively, with an S lens setting of 80.

The mass spectrometer was calibrated in positive mode from 74–1622 m/*z* using Pierce LTQ Velos ESI positive calibration solution (Thermo Fisher Scientific). Comprehensive full-scan and MS/MS data were acquired using the data-dependent acquisition (DDA) scan mode. Full-scan data were collected using the 60,000-resolution setting, an AGC target of 1 × 10^6^ and a max IT of 120 ms. MS/MS data were collected at a resolution of 15,000 with an AGC target of 2 × 10^5^ and an intensity threshold of 5 × 10^4^ for precursor ions, max IT of 100 ms, collision energy (CE) of 20 eV (unless otherwise specified), a loop count of 10 and an isolation window of 0.7 m/*z.* The DDA inclusion list was populated using the masses of reported, detected and predicted ATXs (Table S1), and the exclusion list consisted of all ions detected above the DDA threshold in a blank solvent injection run at the beginning of the sequence. Targeted MS/MS spectra of putative ATXs were collected using the parallel reaction monitoring (PRM) scan mode using a max IT of 3 s with the remaining settings as for DDA. Data analysis was performed manually in Xcalibur 4.0 (Thermo Fisher Scientific).

## Results and discussion

Samples of benthic cyanobacteria associated with dog fatalities on the Wolastoq (Saint John River) in 2018 were originally analysed by LC–HRMS, using typical reverse-phase LC conditions, to confirm the presence of known ATXs [48]. In addition to ATX, H_2_ATX and hATX, these analyses revealed the presence of several other putative ATX analogues (Fig. S2) including addition products of ATX and hATX with thiol-containing biomolecules (GSH, γ-Glu-Cys), or with solvent molecules (H_2_O, CH_3_OH). Conjugates of ATX were hypothesized to occur as a result of nucleophilic attack at the olefinic bond of the α,β-unsaturated ketone (Fig. 1). This is analogous to the thiol conjugation chemistry of MCs, which has been relatively well studied [38–40] and also includes addition of H_2_O and CH_3_OH to MCs [40]. The high pH (e.g. pH ≥ 9) typically observed in late-stage cyanobacterial blooms [45], and reported previously inside benthic cyanobacterial mats [49], mimics conditions often used in Michael addition reactions. An additional significant peak at *m*/*z* 170.1539, consistent with C_10_H_20_NO^+^, was found to co-occur with multiple H_2_ATX isomers at *m*/*z* 168.1383, and was suspected to be the product of reduction of the ketone of H_2_ATX, 10-OH-H_2_ATX (Fig. 1). Since neither thiol conjugates of ATX nor 10-OH-H_2_ATX have been reported previously, we aimed to develop an LC–HRMS/MS method for the comprehensive detection and characterization of ATXs and use a series of semisynthetic reactions from available standards of ATXs to support the identification of new congeners detected.

### Development of LC–HRMS/MS method

**Fig. 2:**
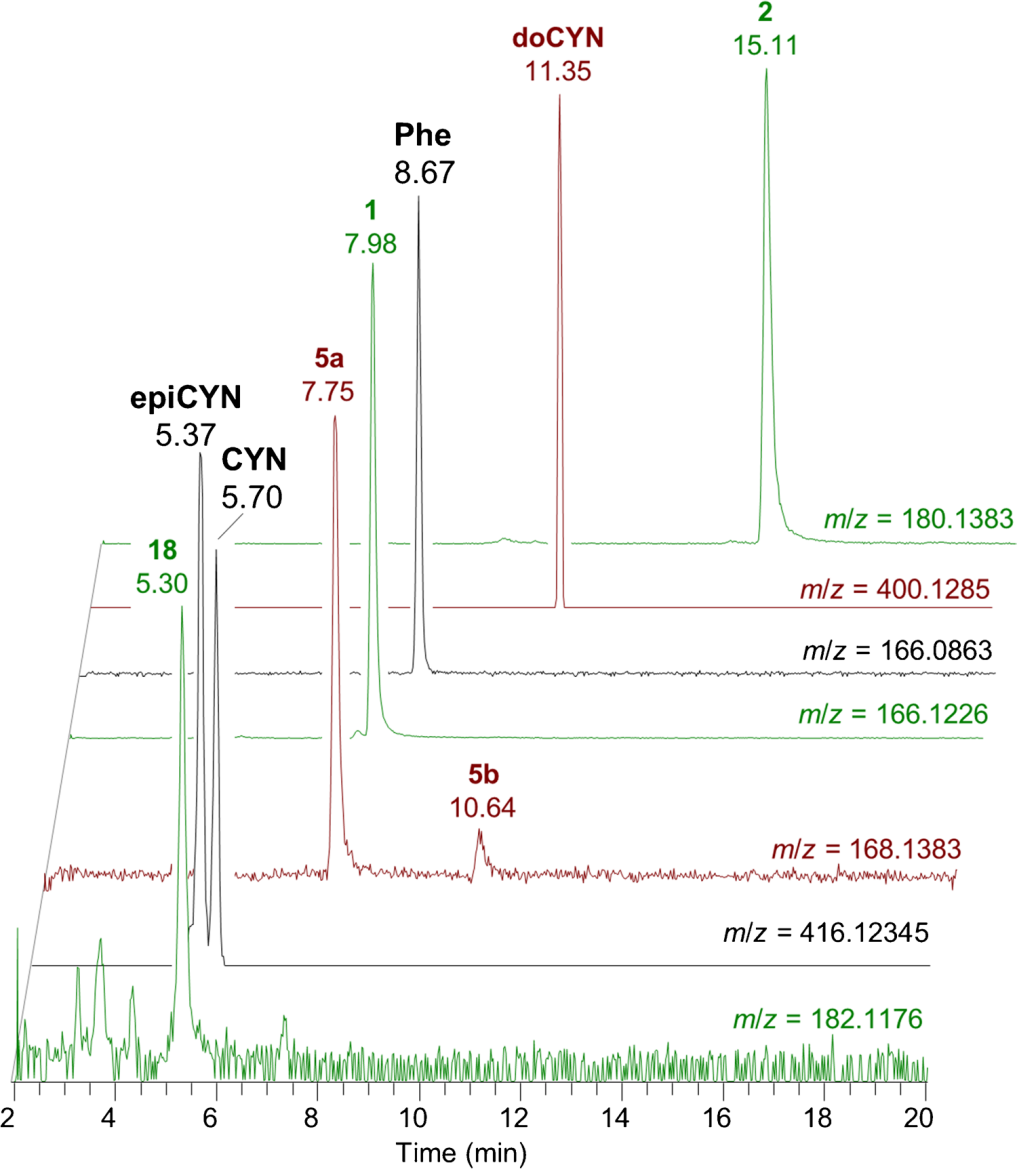
LC–HRMS extracted-ion chromatograms (*m*/*z* ± 3 ppm) of ATX (**1**), hATX (**2**), epoxyATX (**18**), *cis*- and *trans*-H_2_ATX (**5a**, **5b**), CYN, epiCYN, doCYN and phenylalanine (Phe) in RM-BGA

A reverse-phase separation was optimized based on methods previously reported by Foss et al. [50] and Ortiz et al. [51], both of which used a Waters HSS T3 stationary phase and an acidic aqueous acetonitrile mobile phase. In order to improve the retention and resolution of the polar and isomeric ATXs detected, a long, shallow gradient and a longer column with a 1.8-μm particle size were employed. This method showed excellent resolution between ATXs and between ATX and Phe, which is a common source of interference and suppression in MS analysis of ATX [52, 53]. A freeze-dried cyanobacterial matrix reference material (RM-BGA) containing a range of cyanotoxin classes [43] was used to test the separation of known ATXs using the method (Fig. 2). Here, *cis*-H_2_ATX (**5a**) and *trans*-H_2_ATX (**5b**) were differentiated based on their relative retention times using the assignment of Méjean et al. [30]. Though not the focus of the current study, the method was also suitable for analysis of another class of polar cyanotoxins, the cylindrospermopsins. For example, Fig. 2 also shows detection of cylindrospermopsin (CYN), 7-*epi*-cylindrospermopsin (epiCYN) and 7-deoxycylindrospermopsin (doCYN), in RM-BGA.

One limitation of the developed separation, which reflects a general challenge in the analysis of polar analytes by reverse-phase chromatography, was its sensitivity to injections of organic solvent. Because of the low percentage of organic solvent in the mobile phase and the shallow gradient, injections of samples and standards in 1:1 v/v MeOH–H_2_O were typically limited to 1 μL, and injections containing acetonitrile (50% or 75%) to 0.2 μL. Beyond these injection volumes, loss of chromatographic peak shape and resolution were observed. Similarly, the separation was particularly sensitive to small changes in the percentage of the organic mobile phase delivered by the LC system and differences in sample composition, making the method more prone to small variations in retention times between runs (1 to 4% RSD) than typical reverse-phase chromatography methods.

Comprehensive full-scan and HRMS/MS data were acquired using the DDA scan mode, which included source and collision energies optimized for available standards of ATXs. An inclusion list consisting of all known ATXs and a number of potential ATX analogues (Table S1) was used to guide precursor-ion selection in DDA towards ATX-related compounds. The ‘if idle…’ setting was set to ‘pick others’ to allow for the selection of unanticipated ATXs as precursor ions, and an exclusion list based on a blank run was used to minimize MS/MS sampling of background ions.

**Fig. 3:**
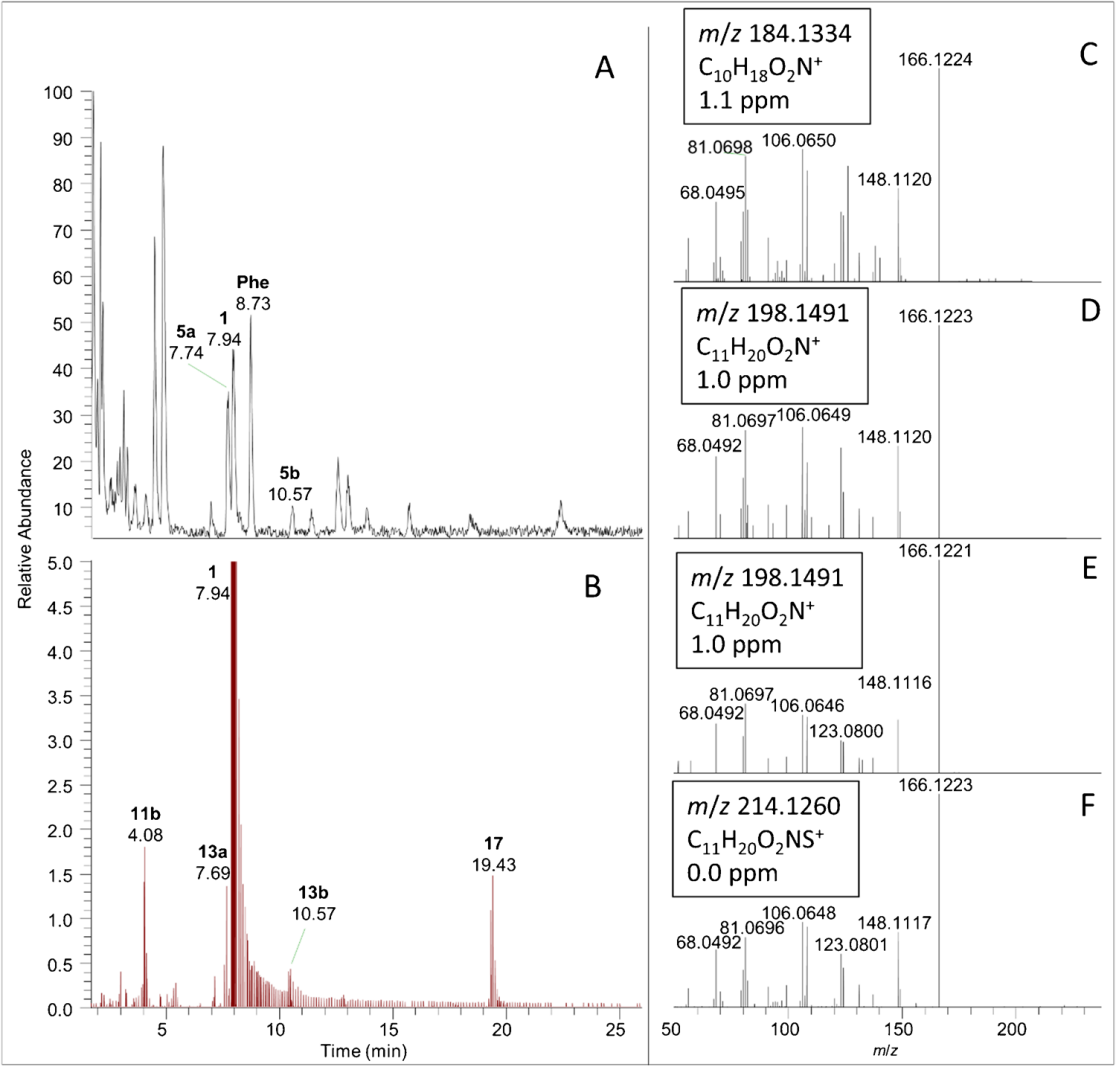
LC–HRMS/MS analysis of a benthic-cyanobacterial-mat field sample in DDA scan mode showing the full-scan base-peak chromatogram with ATX (**1**), *cis-*H_2_ATX (**5a**), *trans-*H_2_ATX (**5b**) and Phe (**A**), and the extracted-ion chromatogram for *m*/*z* 166.1226 ± 5 ppm product ions from all MS/MS data, showing non-target detection of 3-OH-ATX (**11b**), H_3_CO-ATX (**13a**, **13b**) and CH_3_SH-ATX (**17**) (**B**). The product-ion spectra of **11b** (**C**), **13a** (**D**), **13b** (**E**), and **17** (**F**) from the corresponding DDA scans are shown in panes **C**–**F**, with the insets indicating the full-scan accurate mass, corresponding formula, and observed mass error for each precursor-ion

Under optimized source conditions, ATX conjugates showed in-source fragmentation yielding a product ion at *m/z* 166.1226 corresponding to the formula (C_10_H_16_NO^+^), the same as the [M + H]^+^ of ATX. However, the CID spectrum of this fragment differed significantly from that of protonated ATX, suggesting that loss of the conjugating group in the gas phase generates a different isomeric product ion. This is illustrated for 3-OH-ATX in Fig. S3, showing the CID spectrum of the [M + H − H_2_O]^+^ in-source fragment of 3-OH-ATX (Fig. S3D) with predominant loss of H_2_O to yield the *m/z* 148.1126 product ion compared to that of ATX that yielded the *m/z* 149.0961 product ion (Fig. S3E). All detected ATX and hATX conjugates showed similarly facile elimination of the conjugating group in collision-induced dissociation, to form the same *m*/*z* 166.1226 or *m/z* 180.1383 products, respectively. This common fragmentation was exploited for non-targeted detection of conjugates by displaying extracted-ion chromatograms for these product-ion *m*/*z* values using the scan filter in Xcalibur ‘FTMS + p ESI Full ms2’, which includes all CID data collected in a DDA run. This approach is demonstrated in Fig. 3 for a benthic-cyanobacterial-mat sample, which shows DDA detection of 3-OH-ATX (**11**) and two isomers of CH_3_O-ATX (**13a**, **13b**) as well as a new ATX conjugate with methanethiol, CH_3_S-ATX (**17**). The same non-target approach was also applied to an extract of *K. formosum* culture of the strain NIVA-CYA92, which was reported to produce high levels of hATX [19] (Fig. S4). This revealed three peaks at *m*/*z* 244.1146 for isomers of GSH-hATX (**8**) and several peaks at *m*/*z* 184.1332 for 3-OH-hATX (**12**).

**Fig. 4:**
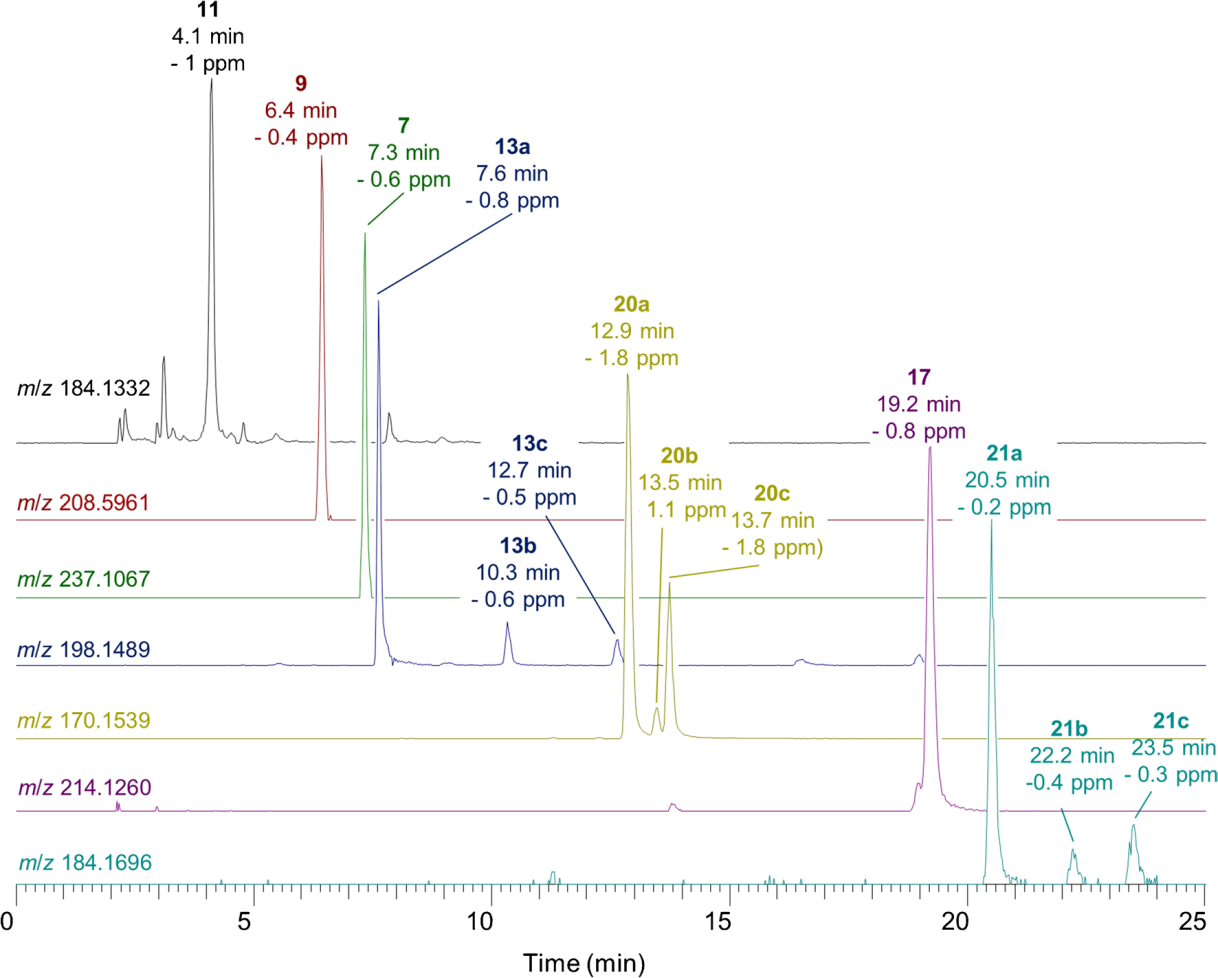
Extracted-ion LC–HRMS chromatograms (± 5 ppm) showing additional ATX analogues detected in LC–HRMS analysis of a benthic-cyanobacterial-mat sample including ATX (**1**), hATX (**2**), H_2_ATX (**5**), H_2_hATX (**6a**, **6b**), GSH-ATX (**7a**), γ-Glu-Cys-ATX (**9b**), 3-OH-ATX (**11b**), CH_3_O-ATX (**13a**, **13b**, **13c**), CH_3_S-ATX (**17**), 10-OH-H_2_ATX (**20a**, **20b**, **20c**), 10-OH-H_2_hATX (**21a**, **21b**, **21c**), 10-OH-ATX (**22a**, **22b**) and 10-OH-hATX (**23a**)

Together, the reverse-phase LC separation and combined full-scan/DDA HRMS/MS detection method showed excellent resolution and sensitive detection of both targeted and non-target ATXs in complex mat samples. Figure 4 shows the detection of over 20 ATXs in a benthic-cyanobacterial-mat field sample, including conjugates and 10-OH analogues. Analysis of an extract of *K. formosum* (Fig. S5) showed the presence of additional hATX analogues. EpoxyATX (**18**) in Fig. 2 and epoxyhATX (**19**) in Fig. S5 were distinguished from hydroxylated analogues based on their product ion at *m*/*z* 140.1070 associated with side-chain elimination during CID [54], which is unique among ATXs to epoxy-ATXs. H_2_hATX (**6**) showed a similar *cis*-/*trans*- epimer pair (**6a** and **6b** in Fig. S5) and product-ion spectra to H_2_ATX. These were differentiated from isomeric 10-OH-ATX and 10-OH-hATXOH based on their CID spectra and semisynthetic confirmation, as described below.

### Confirmation of ATX-variants by semisynthesis

**Scheme 1:**
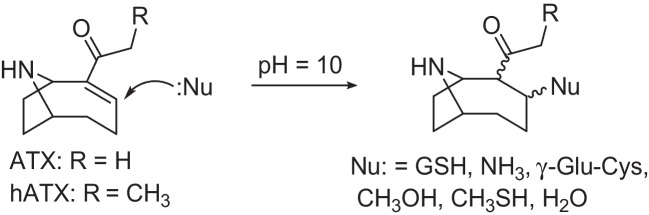
Treatment of an ATX or hATX standard with a conjugating nucleophile under basic conditions to promote Michael addition

**Fig. 5:**
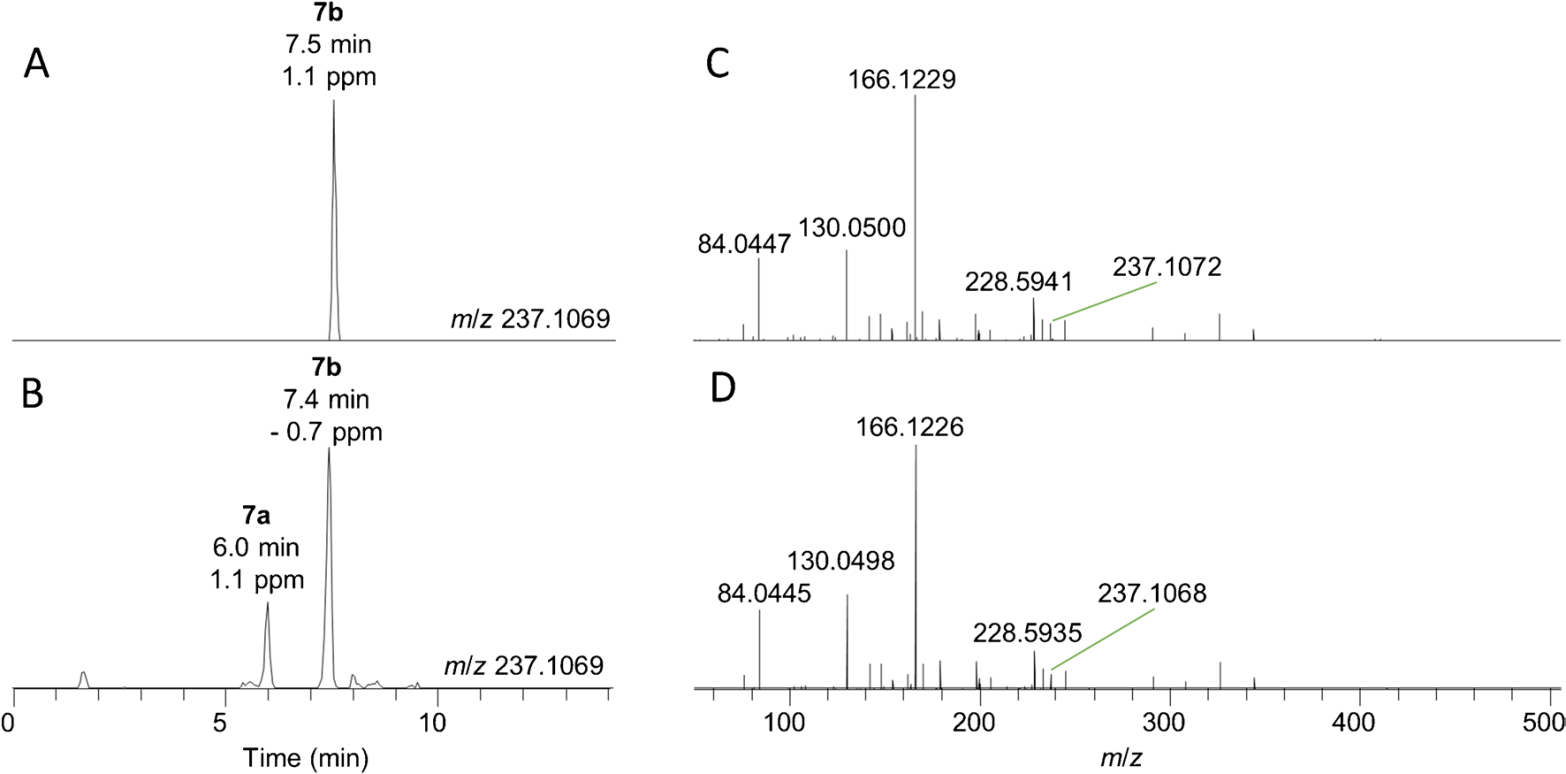
LC–HRMS/MS analysis of GSH-ATX (**7b**) in a benthic-cyanobacterial-mat sample (**A**, **C**) and isomer **7a** and **7b** in a semisynthetic preparation (**B**, **D**) showing extracted-ion chromatograms (exact *m*/*z* ± 5 ppm) (**A**, **B**) and product-ion spectra (**C**, **D**) of **7b** at a CE of 10 eV

Semisynthesis was used to provide confirmation of the structures of new or less commonly reported ATXs. For compounds formed by addition to the α,β-unsaturated ketone, this involved treatment of an ATX or hATX standard with the conjugating compound under basic conditions to promote Michael addition (Scheme 1). An example is shown in Fig. 5 for the conjugation of ATX with GSH. There was good agreement between the full-scan accurate masses, retention times and product-ion spectra of the natural GSH-ATX (**7**) and the main semisynthetic isomer. Semisynthesis also produced an additional abundant GSH-ATX isomer, attributable to one or more of the four possible diastereomers resulting from attack of the thiol nucleophile at the 3-position of ATX (Scheme 1). It is unknown whether the detection of a single GSH-ATX in the field sample is because that isomer is preferentially formed under natural conditions, or whether it is the result of a favourable equilibrium that is achieved over time after initial formation of multiple isomers.

Similar results were obtained for other combinations of ATX with thiol-, hydroxy- and amino-nucleophiles, when compared to cyanobacterial-mat field samples, as shown in Supplementary information for γ-Glu-Cys-ATX (Fig. S6), H_2_N-ATX (Fig. S7), 3-HO-ATX (Fig. S8), CH_3_O-ATX (Fig. S9), and CH_3_S-ATX (Fig. S10). Since the available field samples did not contain high levels of hATX, only low levels of hATX conjugates were detected. However, a mature culture of *K. formosum* strain NIVA-CYA92 that produces high levels of hATX [19] was found to contain several hATX conjugates. These also showed good agreement with the full-scan *m*/*z* and product-ion spectra, and retention times, of semisynthetic hATX conjugates (Supplementary information for GSH-hATX (Fig. S11), γ-Glu-Cys-hATX (Fig. S12), CH_3_O-hATX (Fig. S13), HO-hATX (Fig. S14) and H_2_N-hATX (Fig. S15)). Conjugates of ATX and hATX with Cys and Cys-Gly, as well as the methanethiol conjugate of hATX, were also synthesized, but were not detected in field or culture samples.

**Scheme 2:**
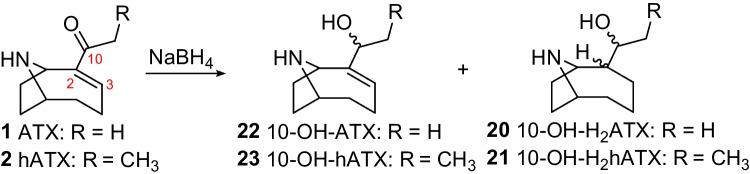
Reduction of ATX and hATX with sodium borohydride

**Scheme 3:**
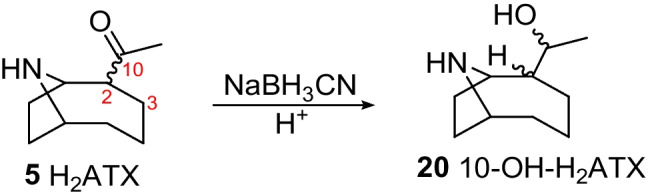
Selective reduction of an H_2_ATX standard using sodium cyanoborohydride

**Fig. 6:**
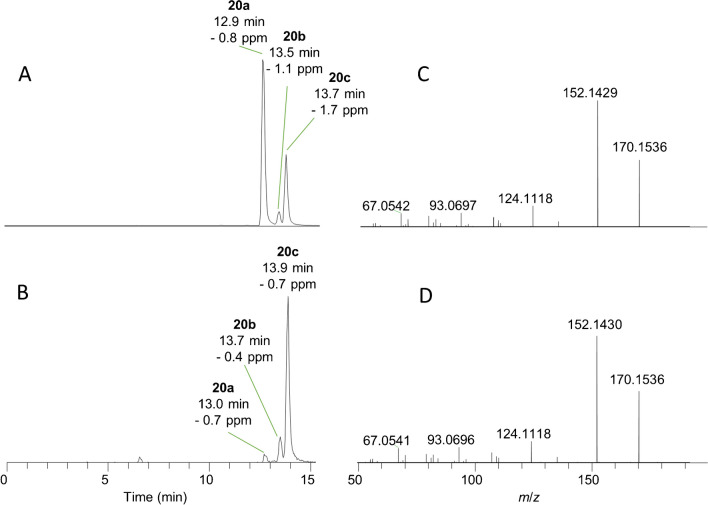
LC–HRMS/MS comparison between natural (**A**, **C**) and semisynthetic (**B**, **D**) 10-OH-H_2_ATX (**20a**, **20b**, **20c**) resulting from treatment of an H_2_ATX standard with sodium cyanoborohydride, showing extracted-ion full-scan chromatograms (**A**, **B**) and product-ion spectra (**C**, **D**) of **20c**

The tentative identification of 10-OH-ATX and 10-OH-H_2_ATX in field samples was supported by chemical reduction of ATX, hATX and H_2_ATX standards. Reduction of ATX and hATX with sodium borohydride yielded a mixture of their 10-OH- and 10-OH-dihydro analogues (Scheme 2), collectively referred to as 10-OH-ATXs. This included a cluster of three closely eluting peaks for 10-OH-H_2_ATX and 10-OH-H_2_hATX that matched in retention time and HRMS/MS spectra with naturally occurring 10-OH-H_2_ATX detected in a benthic-cyanobacterial-mat sample (Fig. S16) and 10-OH-H_2_hATX detected in *K. formosum* culture (Fig. S17). The cluster of peaks presumably represents the four possible isomers arising from the two possible stereochemistries at C-2 and C-10 (Scheme 2). Reactions with sodium borohydride also generated an additional cluster of early-eluting isomers that did not correspond to naturally detected compounds and could represent products of ring opening or rearrangement. Further support for the assignment of structures for **20** and **21** to 10-OH-dihydroanatoxins was obtained from the selective reduction of an H_2_ATX standard using sodium cyanoborohydride (Scheme 3), which yielded only the naturally occurring isomers of 10-OH-H_2_ATX (Fig. 6).

**Fig. 7:**
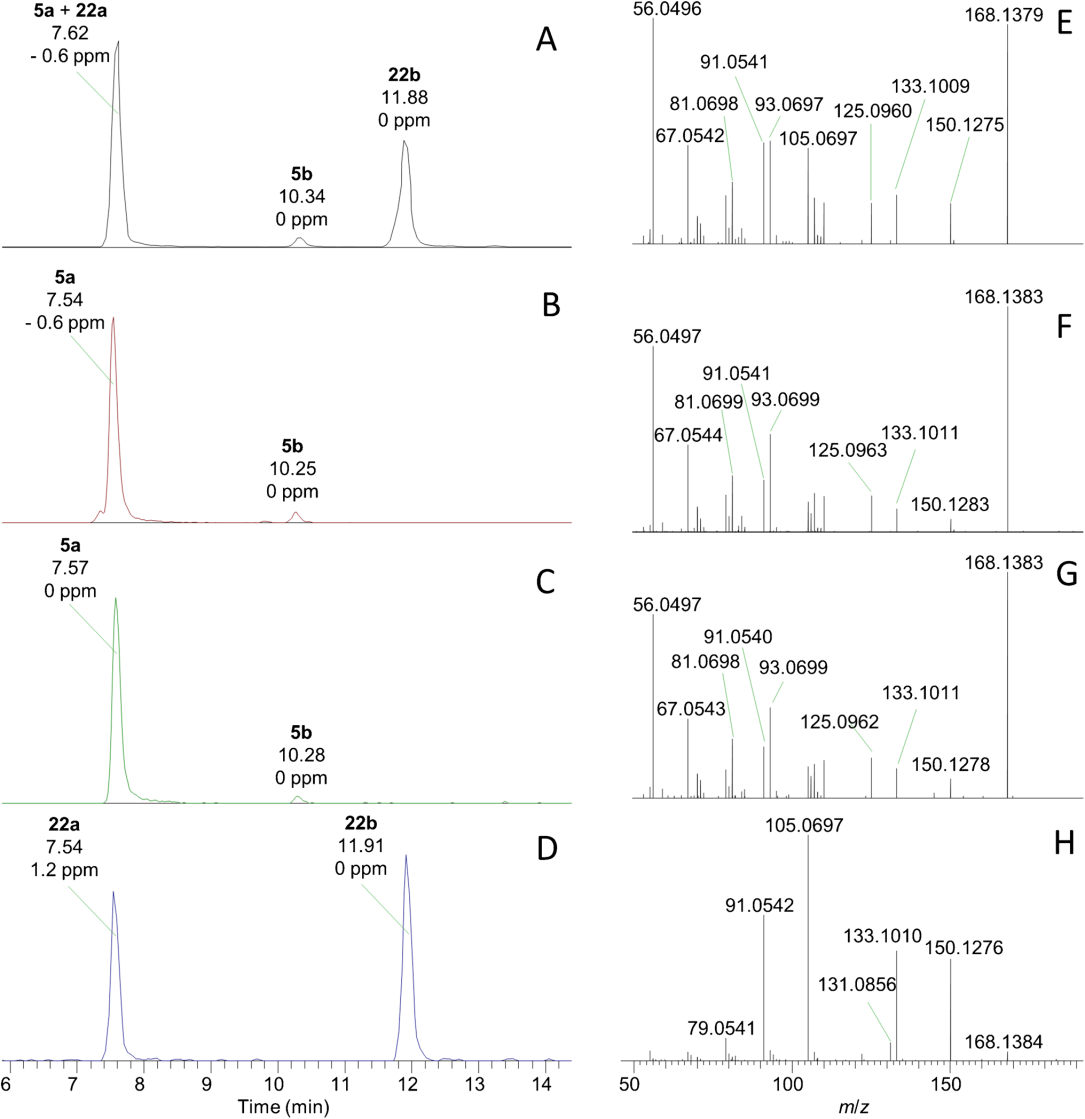
LC–HRMS/MS analysis of isomers of H_2_ATX (**5a**, **5b**) and 10-OH-ATXOH (**22a**, **22b**) in a benthic-cyanobacterial-mat field sample (**A**, **E**), a commercial H_2_ATX standard (**B**, **F**), a laboratory culture *C. issatschenkoi* (**C**, **G**) and the reaction between ATX and sodium borohydride (**D**, **H**) showing extracted-ion chromatograms (*m*/*z* 168.1383 ± 5 ppm) (**A**–**D**) and CID spectra of the *m*/*z* 168.1383 peak at 7.5–7.6 min in each (**E**–**H**)

The semisynthesis of 10-OH-ATX (**20**) and 10-OH-hATX (**21**) by reduction of ATX and hATX standards using sodium borohydride and sodium cyanoborohydride was helpful in differentiating these analogues, which have only been reported previously as synthetic compounds [31], from the commonly observed H_2_ATXs (**5** and **6**). It was found that one isomer of 10-OH-ATX **(22a**) and *cis*-H_2_ATX (**5a**) were not chromatographically resolved using the current method (Fig. 7), but that they could easily be distinguished based on their CID spectra. Figure 7B and F show the chromatogram and CID spectrum of *cis*-H_2_ATX from an isomeric H_2_ATX standard, which was similar between **5a** and **5b**. These matched the compounds found in *C. issatschenkoi* culture (Fig. 7C, G) showing greater precursor-ion stability and relatively little water-loss product at *m*/*z* 150.1277. The products of ATX reduction to 10-OH-ATX (Fig. 7D) both showed CID spectra with almost complete dissociation of the precursor ion and dominant product ions at *m*/*z* 150.1277, 133.1012, 105.06988 and 91.0542 (Fig. 7H). The high-level cyanobacterial-mat field samples analysed contained a mix of all four *m*/*z* 168.1383 isomers (Fig. 7A), and the CID spectrum of the main peak at 7.6 min (Fig. 7E) suggests a mixture of **5a** and **22a**, but dominated by **5a**. Similar results were obtained for 10-OH-hATX and H_2_hATX (Figure S18), but chromatographic resolution was observed between the four *m*/*z* 182.1593 isomers. While *cis*-H_2_hATX (**6a**) was the dominant isomer in the benthic-cyanobacterial-mat field samples analysed (Fig. S18A), this was not the case in *K. formosum* culture (Fig. S18A), which was dominated by 10-OH-hATXOH (**23**).

### Origins of conjugates and reduction products of ATXs

The results of the present study demonstrate that conjugates of ATX and hATX can form via spontaneous chemical reactions at high pH in cyanobacterial blooms and benthic mats [45, 49]. The possibility that glutathione transferase enzymes could be involved in the formation of GSH conjugates detected in the environmental samples must be acknowledged and is one possible explanation for the differences in epimer ratios of conjugates detected between natural and semisynthetic samples; however, there is no direct evidence that this is the case. The conjugation chemistry explored here for ATXs could also play a role in explaining the recently reported discrepancies in the acute toxicities of ATX and H_2_ATX depending on route of exposure [21]. Although Puddick et al. [21] confirmed original reports of the lower toxicity of H_2_ATX by intraperitoneal injection, relative to that of ATX, they found that H_2_ATX was significantly more toxic than ATX by oral administration [21]. The potential for ATX to form conjugates with biomolecules in vivo, thereby reducing its toxicity or rate of uptake, or increasing its rate of excretion, is a possible explanation for this discrepancy in toxicities. In contrast, H_2_ATX lacks the reactive double bond present in ATX, and so is unable to undergo the same type of conjugation reactions. This was supported by the absence of H_2_ATX conjugates in samples analysed in this study, even though some field samples contained very high levels of this compound (up to 148 mg/kg [48]).

In order to shed light on the origin of reaction products of ATX with H_2_O and MeOH, which were detected in field samples that were originally extracted in 50% MeOH [55], sub-samples of a benthic-cyanobacterial-mat were prepared in a variety of other solvents (Fig. S19). Substitution of CH_3_OH for H_2_O, acetonitrile, ethanol or CD_3_OH consistently led to a tenfold decrease in the signal intensity of H_3_CO-ATX at *m*/*z* 198.1489. In the case of extraction with 1:1 v/v H_2_O–CD_3_OH, additional peaks for CD_3_OH addition to ATX were observed at *m/z* 201.1677, with peak areas equivalent to the decrease in peak area of the CH_3_O-ATX addition product. It therefore appears that the majority of the CH_3_O-ATX originally detected in cyanobacterial-mat field samples originated from sample preparation in MeOH, and that only the trace level detected in samples prepared in the absence of MeOH can be attributed to the samples themselves.

Because of the high water content of the field samples analysed, it was more difficult to study the origin of 3-HO-ATX (**11**). Sample extraction in 1:1 v/v H_2_^18^O–H_2_O showed a 30% decrease in the water addition product detected at *m*/*z* 184.1332 and formation of a similar amount (20%) of the addition product of H_2_^18^O to ATX (Fig. S19D). This suggests that at least this much of the 3-HO-ATX originally detected in field samples originated during sample preparation. However, this is likely an underestimate of the true amount, since this reaction will have had a chance to proceed during earlier stages of sampling and sample preparation, prior to extraction. The addition of 0.1% formic acid to the extraction solvent greatly reduced the formation of CH_3_O-ATX during sample preparation (Fig. S20). Since the chromatographic method employed was sensitive to injections containing acetonitrile, 1:1 v/v CH_3_OH–H_2_O with 0.1% formic acid was used for all subsequent sample extractions.

Addition of ammonia to ATX was first detected where samples were prepared in ammonium carbonate buffer, and can also be primarily considered as artefacts of sample preparation. Figure S7 shows only trace detection of H_2_N-ATX when a cyanobacterial-mat field sample was prepared in 1:1 v/v MeOH–H_2_O, but significant conversion (50% peak area relative to ATX) when ammonia was included in the extraction buffer. Similar addition of ammonia to the α,β-unsaturated amide of MCs has also recently been observed in our laboratory (manuscript in preparation). It is therefore recommended that ammoniated buffers and mobile phases be avoided in sample preparation and analysis of cyanotoxins.

**Fig. 8:**
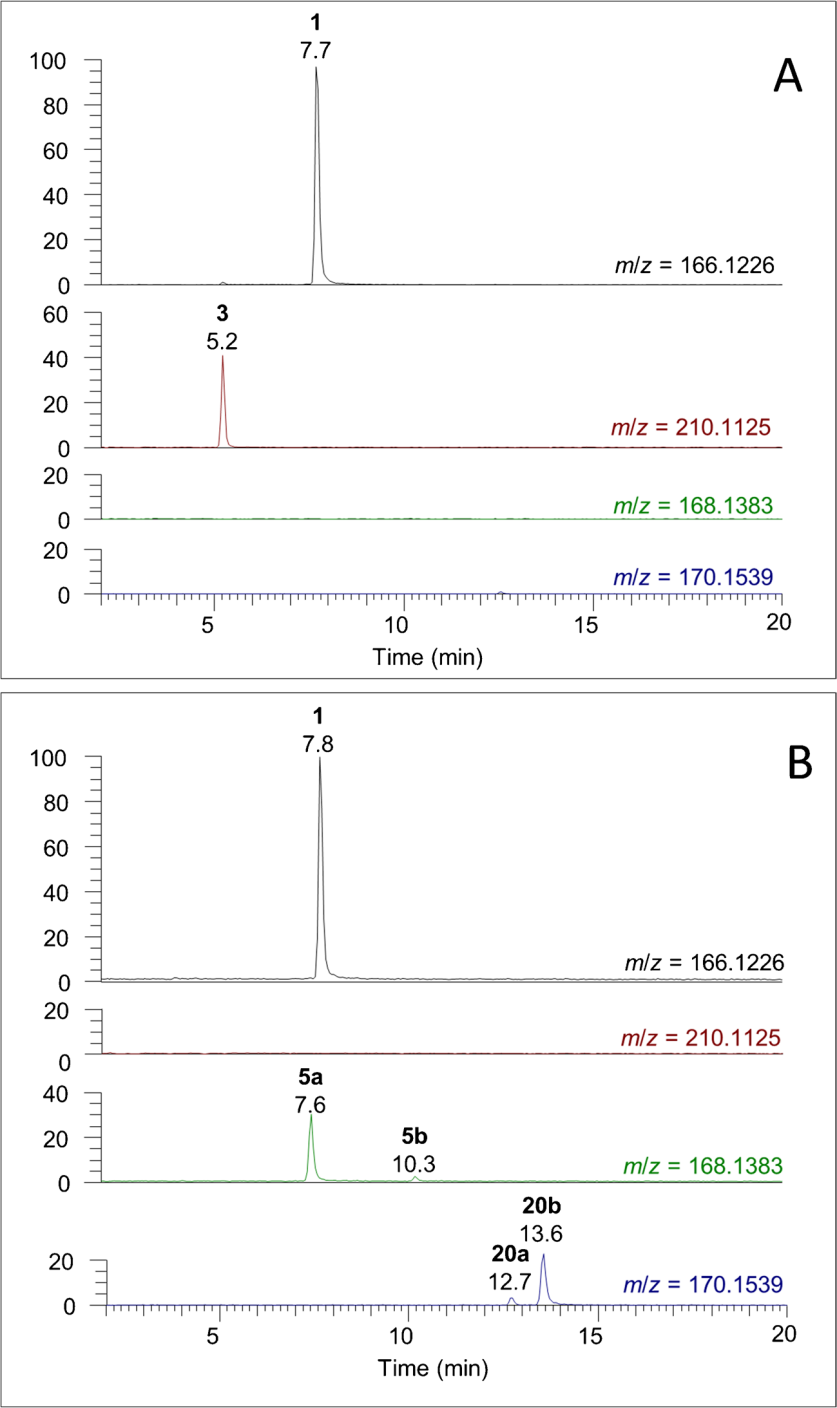
Extracted-ion chromatograms from LC–HRMS analysis of preparations of *C. issatschenkoi* with acidic MeOH extraction: before (**A**) or after (**B**) freeze–thaw cell lysis, showing ATX (**1**) in both samples and carboxyATX (**3**) in the sample extracted prior to cell lysis, as well as *cis*-H_2_ATX (**5a**), *trans*-H_2_ATX (**5b**) and at least two isomers of 10-OH-H_2_ATX (**20a**, **20b**) in the sample extracted after cell lysis. All traces are shown on the same relative scale

Since reduction chemistry would not be expected to occur spontaneously in the environment or during sample preparation, it is likely that the 10-OH-ATXs detected originate from cyanobacteria. The detection of 10-OH-H_2_ATX in *C. issatschenkoi* (Fig. 8) as well as 10-OH-hATX and 10-OH-H_2_hATX in culture extracts of *K. formosum* (Fig. S21) further supports this notion. However, it was observed that 10-OH-H_2_ATXs were detected at significantly lower levels when cultured cyanobacteria were extracted with 1:1 v/v CH_3_OH–H_2_O containing 0.1% formic acid prior to cell lysis (Fig. 8 and Fig. S21), a sample treatment that was reported previously to inactivate the decarboxylase enzymes responsible for converting the carboxyATX precursor to ATX [24]. Surprisingly, a similar trend was observed for H_2_ATX (**5**) in *C. issatschenkoi* and 10-OH-hATX (**23**) in *K. formosum*, which were also found only in relatively small amounts in culture samples extracted prior to cell lysis, compared to samples that had been lysed by freeze–thaw prior to extraction with MeOH (Fig. 8 and Fig. S21). H_2_ATX was originally considered a degradation product of ATX [22], but is now believed to be a biosynthetic product of an F_420_ reductase enzyme coded by the *anaK* gene [30]. Our results suggest that while production of H_2_ATXs and 10-OH-ATXs is enzymatic, in the strains and under the conditions studied here, it is catalyzed by enzymes released during cell lysis rather than being solely associated with the *ana* gene cluster. Differential formation of H_2_ATX (**5**) in *C. issatschenkoi* and 10-OH-hATX (**23**) in *K. formosum* suggests that enzymatic reactions occurring outside of the cell that are capable of transforming ATXs differ between species and should be a topic of more detailed study in the future.

### Occurrence of ATX conjugates and 10-OH-ATXs in cyanobacterial samples

To help assess the potential environmental and public heath importance of the conjugated forms of ATXs and 10-OH-ATXs identified in this study, a set of cyanobacterial samples was screened using the developed methodology. Samples included the two strains of laboratory-cultured cyanobacteria, a cyanobacterial matrix reference material previously characterized for cyanotoxins [43] and 22 benthic-cyanobacterial-mat samples from NB, Canada, that had previously been analysed for ATX, hATX and H_2_ATX as part of recent studies [12, 48, 55, 56]. Samples ranged in total targeted ATXs between < LOD (~ 0.1 μg/kg) to 155 mg/kg, and were generally dominated by ATX and H_2_ATX, with the exception of the *K. formosum* culture, which produced predominantly hATX analogues.

In the absence of analytical standards or LC–MS response factors for most analogues, relative peak areas were used to estimate contributions to total ATXs (Table S2). Conjugates were detected in samples with high concentrations of targeted ATXs, but less so in lower-level ones. The summed peak area of all conjugates relative to that of all ATXs ranged between 0.9 and 15% in samples where they were detected, with an average of 4 ± 5% (standard deviation). Thiol conjugates were relatively low in the field samples compared with H_2_O or CH_3_OH addition products. By comparison, the *K*. *formosum* strain showed a much higher proportion of thiol conjugates, including GSH-hATX at 10% of the peak area of all ATXs.

10-OH-anatoxins were more consistently detected, and at higher levels than for the conjugates in both environmental samples and cultured cyanobacteria. Levels of 10-OH-H_2_ATX ranged from 1.6 to 37% relative to total ATXs in positive samples, with an average of 15 ± 10% (standard deviation). 10-OH-ATXs were also detected in both strains of cultured cyanobacteria, though the level of 10-OH-H_2_hATX in *K. formosum* was relatively low (0.6%) compared to that of 10-OH-H_2_ATX in *C. issatschenkoi* (18%). Conversely, 10-OH-hATX was detected in very high proportions (59% of total ATXs) in the *K. formosum* culture extract. Figure S22 shows the LC–HRMS peak area profiles of the field sample set across multiple sampling sites (Fig. S22A) and across multiple sampling days (Fig. S22B), demonstrating that, in general, conjugates and 10-OH-ATXs co-occurred with targeted ATXs and showed similar spatiotemporal trends. Overall, conjugates and 10-OH-ATXs accounted for up to 52% of total ATXs in benthic-cyanobacterial-mat field samples and 74% in the *K. formosum* culture.

Several aspects should be addressed in future work aimed at assessing the potential toxicity and risk of the broader suite of anatoxins detected in this study. Given that both secondary amine and side-chain carbonyl functionality are required for ATX toxicity [32], it is not expected that 10-OH-ATXs would be highly toxic, which has been supported experimentally in the case of 10-OH-ATX [31]. However, the origin and potential transformations of 10-OH-ATXs should still be studied, including their chemical and biochemical relationships with highly toxic ATXs and dihydroanatoxins. For example, oxidation of the 10-OH group of 10-OH-H_2_ATX could also occur in vivo, to give highly toxic H_2_ATXs. In the case of ATX conjugates, there exists a significant potential for de-conjugation chemistry, which would result in their transformation back to toxic parent ATXs, which could result in an underestimation of toxicity in cases where conjugates are not considered. Additionally, there is a potential for ATXs to be conjugated to nucleophilic residues of proteins in food or in tissues in vivo, forming a difficult-to-detect reservoir of potentially releasable ATXs.

## Conclusions

This work describes the development and application of a comprehensive LC–HRMS/MS method for analysis of ATXs, including a strategy for non-target analysis of new conjugates. We report the detection of several new conjugates and 10-OH-ATXs in field samples of benthic cyanobacteria and laboratory-cultured strains. Tentative identifications based on HRMS/MS data were supported by semisynthetic preparation of all new analogues from available standards of ATXs. Based on the chemical reactivity of anatoxins at high pH, it is expected that the detected conjugates result from chemical reactions in the environment or during sample preparation. In laboratory cultures, 10-OH-ATXs were found to originate primarily from enzymatic transformations that occurred after cell lysis. Further work is required to determine how widespread these transformations are among cyanobacterial strains and in the environment.

Finally, the developed methodology was applied to a set of 22 field samples of benthic cyanobacterial mats from NB, Canada, to examine the occurrence of conjugates and 10-OH-ATXs. This showed conjugates to have a relatively minor contribution to total ATXs and to be detected primarily in higher-level samples. On the other hand, 10-OH-H_2_ATX represented a significant proportion of the total ATXs measured and occurred at levels up to 130-fold higher than ATX itself. Future work on the origin, fate and toxicity of 10-OH-ATXs, as well as the potential for de-conjugation of ATX conjugates, is recommended in order to better assess the potential risks to human and environmental health of the broader suite of ATXs reported in this study.

## Supplementary information

Below is the link to the electronic supplementary material.

Supplementary file1 (PDF 960 KB)
