# Supplementary material for: Liquid chromatography–high-resolution tandem mass spectrometry of anatoxins, including new conjugates and reduction products
Source: Anal Bioanal Chem. 2023 Jul 29;415(22):5281–96. doi: 10.1007/s00216-023-04836-y (PMC10444699; doi:10.1007/s00216-023-04836-y)
Supplement: Supplementary file 1 — Supplementary file1 (PDF 960 KB) [file 216_2023_4836_MOESM1_ESM.pdf]

Liquid Chromatography–High-Resolution Tandem Mass Spectrometry of Anatoxins, Including New Conjugates and Reduction Products

Daniel G. Beach,* Lydia Zamlynny, Melanie MacArthur, Christopher O. Miles

Biotoxin Metrology, National Research Council Canada, 1411 Oxford St., Halifax, N.S., Canada

*Corresponding author:
Dr. Daniel Beach
Tel.: +1 (902) 426-8274
E-mail address: daniel.beach@nrc-cnrc.gc.ca

Table of Contents **page #**

Figure S1: Structures, abbreviations, and exact *m/z* of ATXs S2
Table S1: Inclusion list for *m/z* values prioritized for MS/MS selection in DDA S3
Figure S2: Preliminary LC-HRMS analysis of a benthic cyanobacterial mat sample S4
Figure S3: LC–HRMS/MS analysis of 3-OH-ATX S5
Figure S4: Data dependent LC–HRMS/MS analysis of *Kamtonema formosum* S6
Figure S5: LC–HRMS analysis of typically reported anatoxins in *K. formosum* S7
Figure S6: LC–HRMS/MS analysis of natural and semi-synthetic γ-Glu-Cys-ATX S8
Figure S7: Extracted ion chromatograms for H_2N-ATX in a cyanobacterial mat S9
Figure S8: LC–HRMS/MS analysis of natural and semisynthetic 3-OH-ATX S10
Figure S9: LC–HRMS/MS analysis of natural and semisynthetic CH_3O-ATX S11
Figure S10: LC–HRMS/MS analysis of natural and semisynthetic CH_3S-ATX S11
Figure S11: LC–HRMS/MS analysis of natural and semisynthetic GSH-hATX S12
Figure S12: LC–HRMS/MS analysis of natural and semisynthetic γ-Glu-Cys-hATX S13
Figure S13: LC–HRMS/MS analysis of natural and semisynthetic CH_3O-hATX S14
Figure S14: LC–HRMS/MS analysis of natural and semisynthetic 3-OH-hATX S15
Figure S15: LC–HRMS/MS analysis of natural and semisynthetic H_2N-hATX S16
Figure S16: LC–HRMS/MS analysis of natural and semisynthetic 10-OH-H_2ATX S17
Figure S17: LC–HRMS/MS analysis of natural and semisynthetic 10-OH-H_2hATX S18
Figure S18: LC–HRMS/MS analysis of H_2hATX and 10-OH-hATX S19
Figure S19: LC–HRMS analysis of samples prepared in different solvents S20
Figure S20: LC–HRMS analysis of samples prepared with and without acid S21
Figure S21: LC–HRMS analysis of *K. formosum* extracted before and after cell lysis S22
Table S2: Relative abundance of ATXs in cyanobacterial samples S23
Figure S22: Anatoxin profiles in benthic cyanobacterial mats from the Wolastoq S24
References

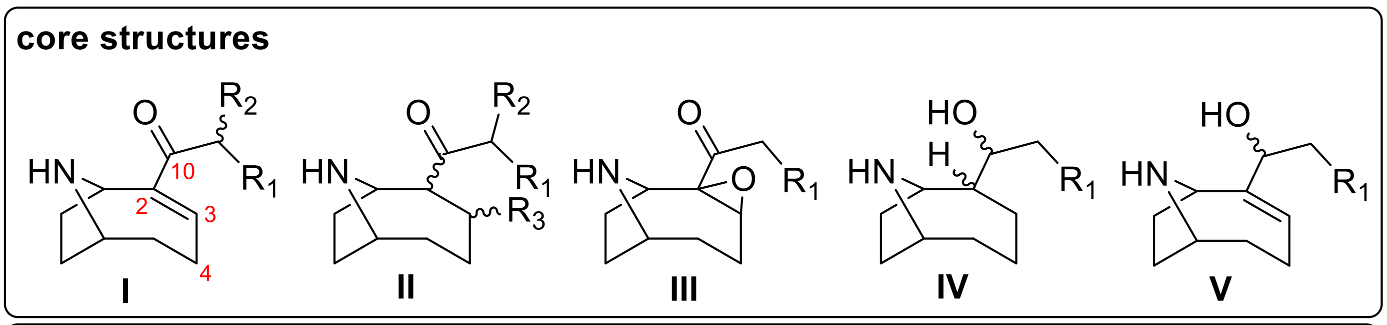

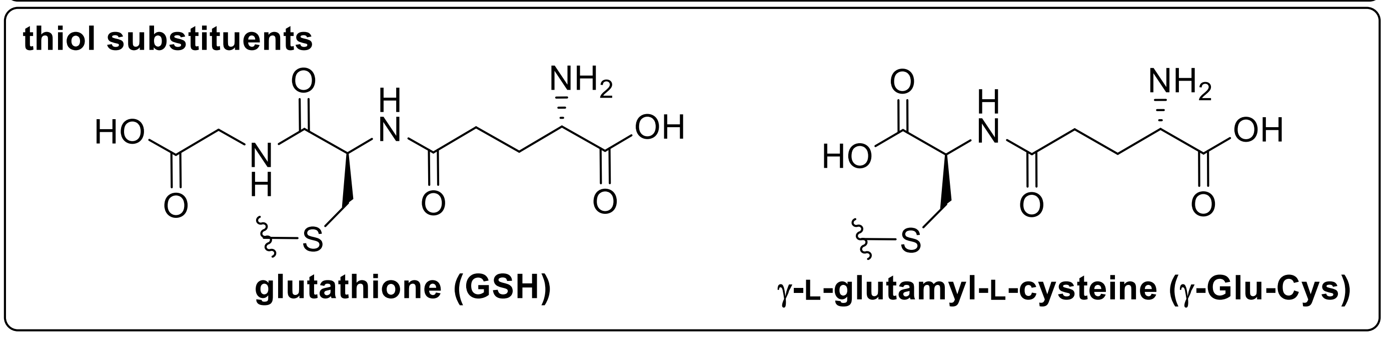

Compound #	Abbrev.	Core	R_1	R_2	R_3	Formula	*m/z*	Isomer #	Retention Time (min)
**1**	ATX	I	H	H	-	$C_{10}H_{16}NO^+$	166.1226	**1**	7.9
**2**	hATX		CH_3	H	-	$C_{11}H_{18}NO^+$	180.1383	**2**	14.9
**3**	carboxyATX		H	COOH	-	$C_{11}H_{17}NO_3^+$	210.1125	**3**	5.2
**4**	carboxyhATX		CH_3	COOH	-	$C_{12}H_{18}NO_3^+$	224.1281	**4**	12.6
**5**	*cis*-H_2ATX	II	H	H	H	$C_{10}H_{18}NO^+$	168.1383	**5a**	7.7
	trans-H_2ATX							**5b**	10.4
**6**	*cis*-H_2hATX		CH_3	H	H	$C_{11}H_{20}NO^+$	182.1539	**6a**	14.2
	trans-H_2hATX							**6b**	19.7
**7**	GSH-ATX		H	H	GSH	$C_{20}H_{34}N_4O_7S^{2+}$	237.1069	**7a**	6.0
								**7b**	7.4
**8**	GSH-hATX		CH_3	H	GSH	$C_{21}H_{36}N_4O_7S^{2+}$	244.1147	**8a**	6.8
								**8b**	9.6
								**8c**	10.5
								**8d**	13.1
**9**	γ-Glu-Cys-ATX		H	H	γ-Glu-Cys	$C_{18}H_{31}N_3O_6S^{2+}$	208.5961	**9a**	5.4
								**9b**	6.4
								**9c**	6.9
**10**	γ-Glu-Cys-hATX		CH_3	H	γ-Glu-Cys	$C_{19}H_{33}N_3O_6S^{2+}$	215.6040	**10a**	6.1
								**10b**	8.3
								**10b**	9.6
								**10d**	11.8
**11**	3-OH-ATX		H	H	HO	$C_{10}H_{18}NO_2^+$	184.1332	**11a**	3.0
								**11b**	4.0
								**11c**	4.6
								**11d**	5.4
**12**	3-OH-hATX		CH_3	H	HO	$C_{11}H_{20}NO_2^+$	198.1489	**12a**	4.8
								**12b**	7.5
								**12c**	8.6
**13**	CH_3O-ATX		H	H	CH_3O	$C_{11}H_{20}NO_2^+$	198.1489	**13a**	7.5
								**13b**	10.3
								**13c**	12.6
**14**	CH_3O-hATX		CH_3	H	CH_3O	$C_{12}H_{22}NO_2^+$	212.1645	**14a**	4.8
								**14b**	7.5
								**14c**	8.6
**15**	NH_2-ATX		H	H	H_2N	$C_{10}H_{17}N_2O^+$	183.1492	**15**	1.8
**16**	NH_2-hATX		CH_3	H	H_2N	$C_{11}H_{19}N_2O^+$	197.1648	**16**	1.8
**17**	CH_3SH-ATX		H	H	CH_3S	$C_{11}H_{20}NOS^+$	214.1260	**17**	19.1
**18**	epoxyATX	III	H	-	-	$C_{10}H_{16}NO_2^+$	182.1176	**18**	5.3
**19**	epoxyhATX		CH_3	-	-	$C_{11}H_{18}NO_2^+$	196.1332	**19**	10.8
**20**	10-OH-H_2ATX	IV	H	-	-	$C_{10}H_{20}NO^+$	170.1539	**20a**	12.8
								**20b**	13.4
								**20c**	13.6
**21**	10-OH-H_2hATX		CH_3	-	-	$C_{11}H_{22}NO^+$	184.1696	**21a**	20.3
								**21b**	22.0
								**21c**	23.2
**22**	10-OH-ATX	V	H	-	-	$C_{10}H_{18}NO^+$	168.1383	**22a**	7.7
								**22b**	12.0
**23**	10-OH-hATX		CH_3	-	-	$C_{11}H_{20}NO^+$	182.1539	**23a**	13.3
								**23b**	20.9

Figure S1: Structures, abbreviations, and exact *m/z* of ATXs ($[M+H]^+$ or $[M+2H]^{2+}$, as indicated) detected in field and culture samples in this study. *Cis*- (**5a**, **6a**) and *trans*- (**5b**, **6b**) isomers of H_2ATX (**5**) and H_2hATX (**6**) are defined based on assignments in Méjean et al. [1]. Isomer numbers were assigned sequentially based on chromatographic elution order. Retention times presented are average values and typically varied by between 1% to 4% relative standard deviation between runs.

Table S1: Inclusion list for *m/z* values prioritized for MS/MS selection in data dependent acquisition LC-HRMS/MS.

name	*m/z*	*z*
Cys-ATX	143.5709	2
Cys-hATX	150.5788	2
ATX	166.1226	1
N-acetylCys-ATX	165.0801	2
H_2ATX-ATX	167.1307	2
H_2ATX	168.1383	1
10-OH-H_2ATX	170.1539	1
Cys-Gly-ATX	172.0817	2
N-acetylCys-hATX	172.0882	2
ATX-hATX	173.1307	2
hATX-H_2ATX	174.1386	2
Cys-Gly-hATX	179.5934	2
oxoATX	180.1019	1
hATX	180.1383	1
epoxy/hydroxyATX	182.1176	1
H_2hATX	182.1539	1
H_2N-ATX	183.1492	1
3-OH-ATX	184.1332	1
10-OH-H_2hATX	184.1696	1
3,10-diOH-ATX	186.1489	1
CN-ATX	193.1335	1
oxohATX	194.1176	1
hATX +CH_2	194.1539	1
epoxy/hydroxyhATX	196.1330	1
H_2N-hATX	197.1648	1
CH_3O-ATX / 3-OH-hATX	198.1489	1
HS-ATX	200.1104	1
2,3-diOH-ATX	200.1281	1
3,10-diOH-hATX	200.1645	1
$(H_2O)_2$-ATX	202.1438	1
CN-hATX	207.1492	1
γ-Glu-Cys-ATX	208.5961	2
carboxyATX	210.1125	1
carboxyH_2ATX	212.1281	1
CH_3O-hATX	212.1645	1
CH_3S-ATX / HS-hATX	214.1260	1
2,3-diOH-hATX	214.1438	1

name	*m/z*	*z*
γ-Glu-Cys-hATX	215.6040	2
$(H_2O)_2$-hATX	216.1594	1
pinnamine	222.1489	1
carboxyhATX	224.1281	1
carboxyH_2hATX	226.1438	1
CH_3S-ATX	228.1417	1
carboxyH_4hATX	228.1594	1
GSH-ATX	237.1069	2
cysteamine-ATX	243.1526	1
GSH-hATX	244.1147	2
cysteamine-hATX	257.1682	1
mercaptoacetic acid-ATX	258.1158	1
mercaptoacetic acid-hATX	272.1315	1
Cys-ATX	287.1424	1
Cys-hATX	301.1580	1
N-acetylCys-ATX	329.1530	1
ATX-ATX	331.2380	1
ATX-H_2ATX	333.2537	1
N-acetylCys-hATX	343.1686	1
Cys-Gly-ATX	344.1639	1
ATX-hATX	345.2537	1
hATX-H_2ATX	347.2693	1
Cys-Gly-hATX	359.1873	1
hATX-hATX	359.2693	1
γ-Glu-Cys-ATX	417.1928	1
γ-Glu-Cys-hATX	431.2085	1
GSH-ATX	474.2143	1
GSH-hATX	488.2299	1

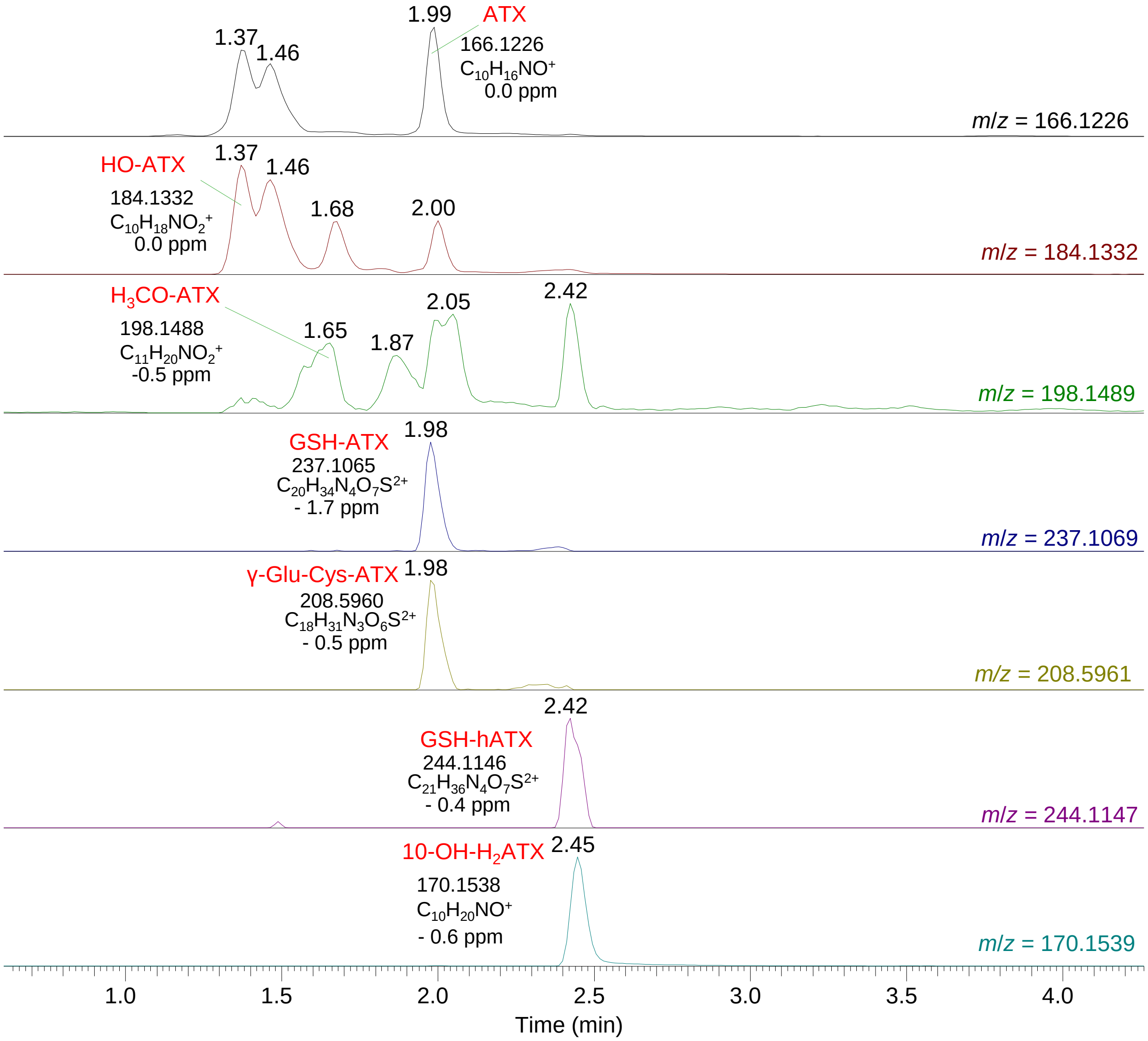

Figure S2: Extracted ion chromatograms (± 5 ppm) from preliminary LC–HRMS analysis of a benthic cyanobacterial mat sample showing detection of putative anatoxin-a (ATX) and homoanatoxin-a (hATX) conjugates with water, methanol, glutathione (GSH) and γ-glutamylcystine (γ-Glu-Cys) as well as 10-OH-H_2ATX. Method details previously published in McCarron et al 2023.[2]

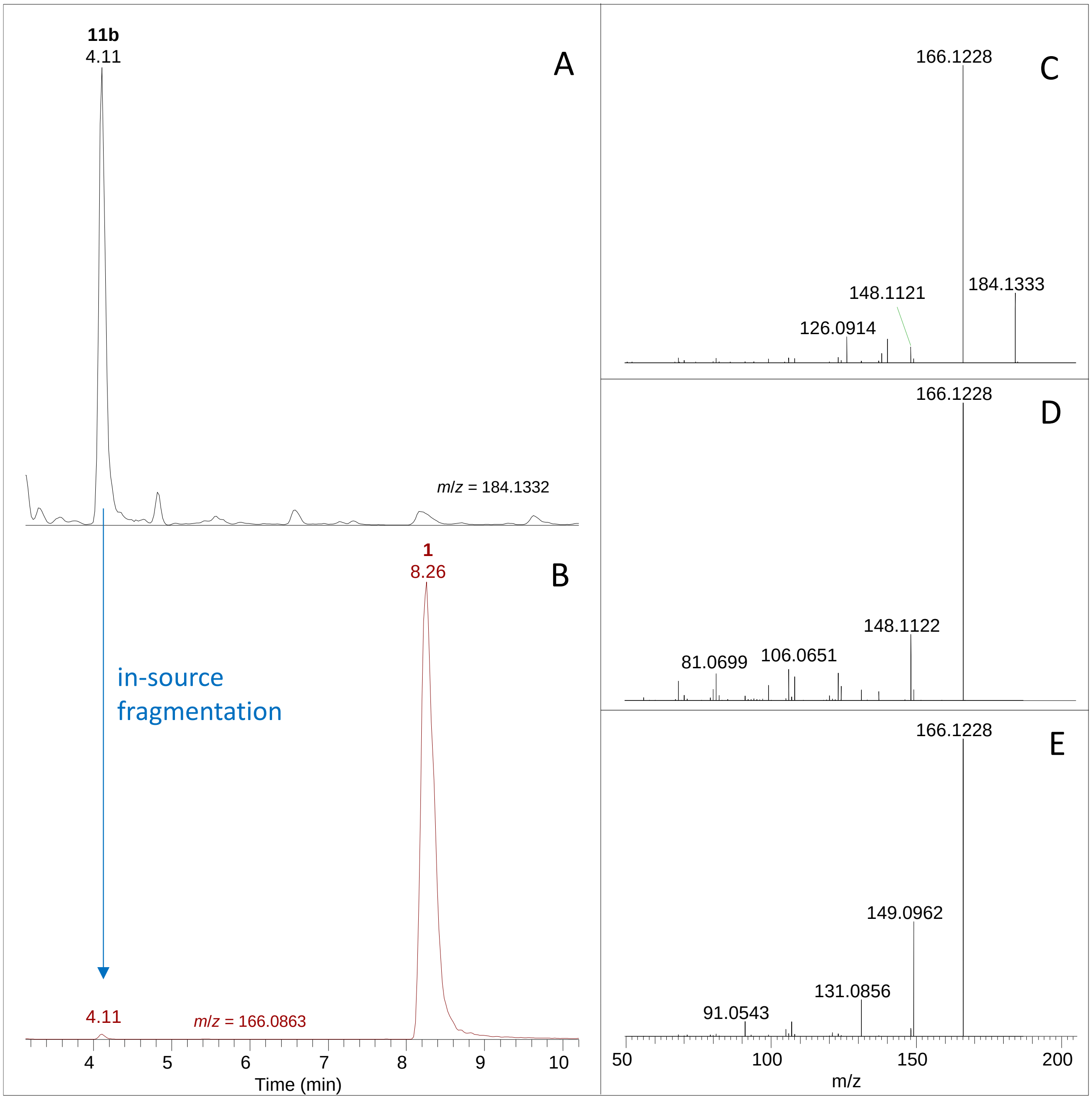

Figure S3: LC–HRMS/MS analysis of 3-OH-ATX (**11b**) including extracted ion chromatograms (*m/z* ± 3 ppm) of the $[M+H]^+$ ion at *m/z* 184.1332 (A) as well as ATX (**1**) and the water-loss source fragment of 3-OH-ATX at *m/z* 166.1226 (B). Collision-induced dissociation spectra of **11b** (C), the water loss source fragment of **11b** (D) and of **1** (E), collected at CE = 10 eV.

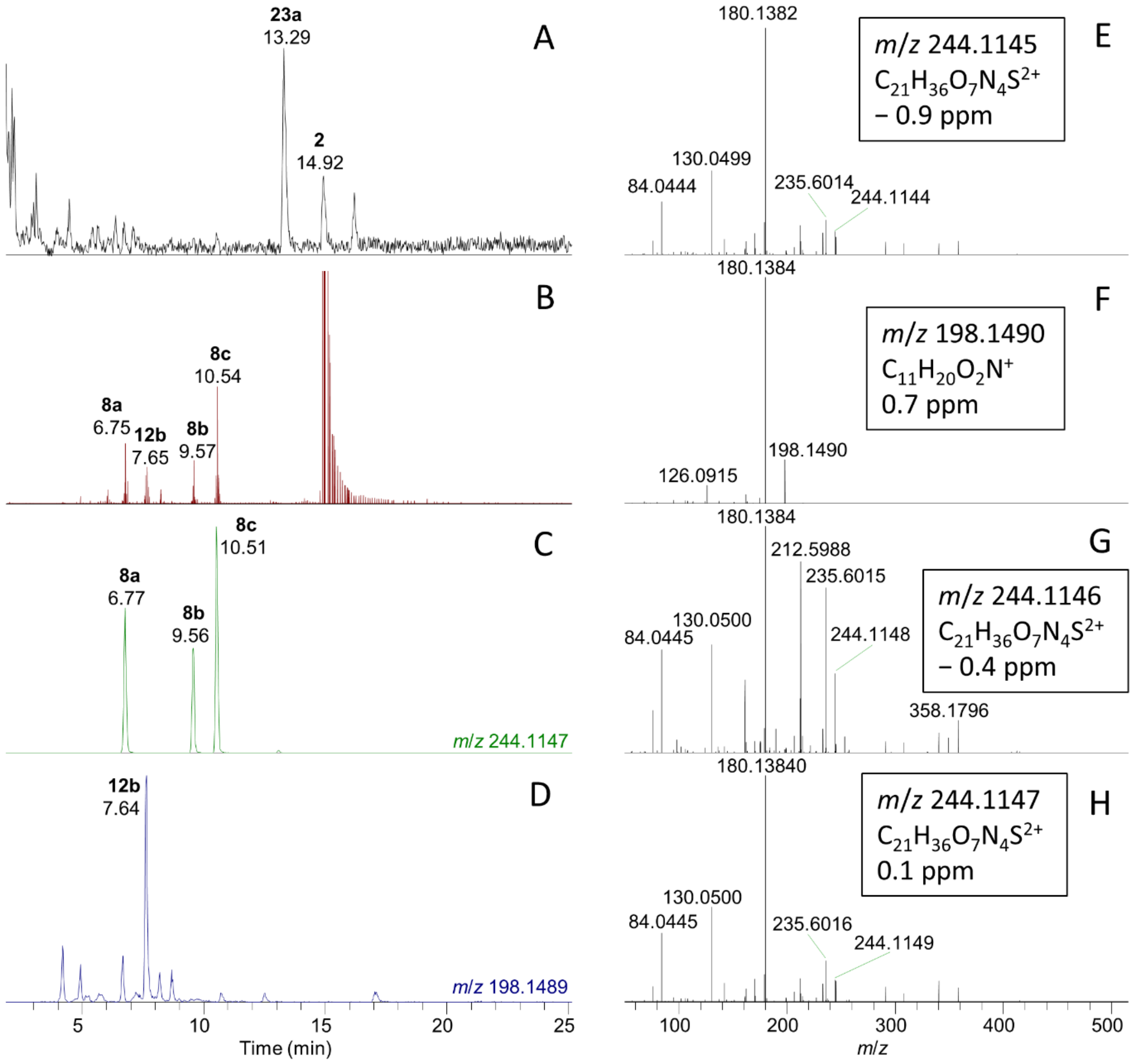

Figure S4: LC–HRMS/MS analysis of *K. formosum* in data-dependent acquisition (DDA) scan mode showing the full-scan total ion chromatogram with hATX (**2**), H_2hATX (**6**) and 10-OH-hATXOH (**23a**) peaks annotated (A) and the extracted ion chromatograms (± 5 ppm) for the *m*/*z* 180.1383 product ion from all MS/MS data showing non-target detection of isomers of GSH-hATX (**8a**, **8b**, **8c**, pane C) and 3-OH-hATX (**12b**, pane D). Product ion spectra of **8a** (E), **12** (F), **8b** (G) and **8c** (H) are from corresponding DDA scans at CE = 10 eV. Insets on panes E-H show the full-scan exact mass, corresponding ionic formula and mass error for each precursor.

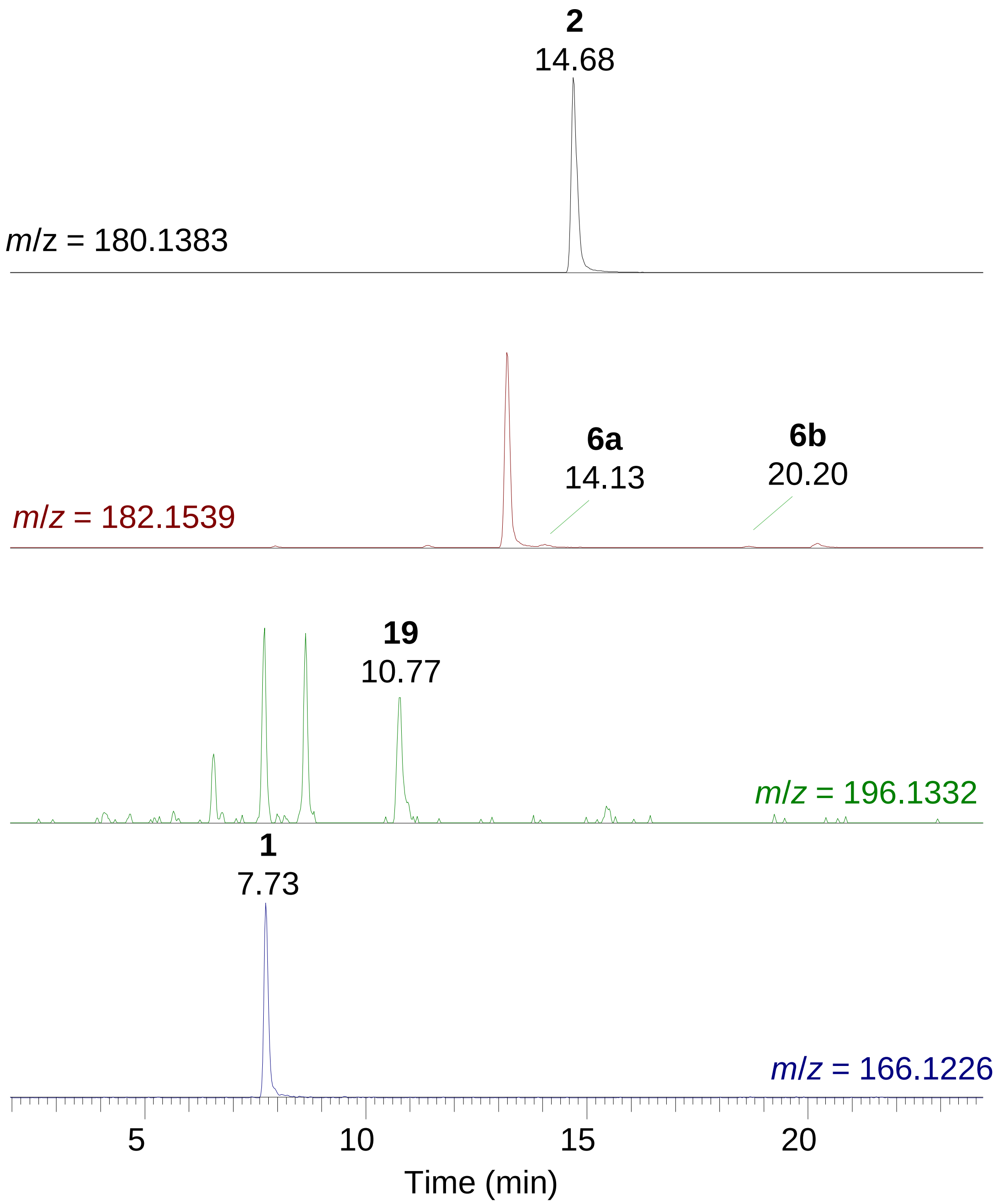

Figure S5: LC–HRMS analysis of known ATXs in an extract of *K. formosum*, including anatoxin-a (**1**), homoanatoxin-a (**2**), epoxyhomoanatoxin-a (**19**) as well as two isomers of dihydrohomoanatoxin-a (**6a, 6b**).

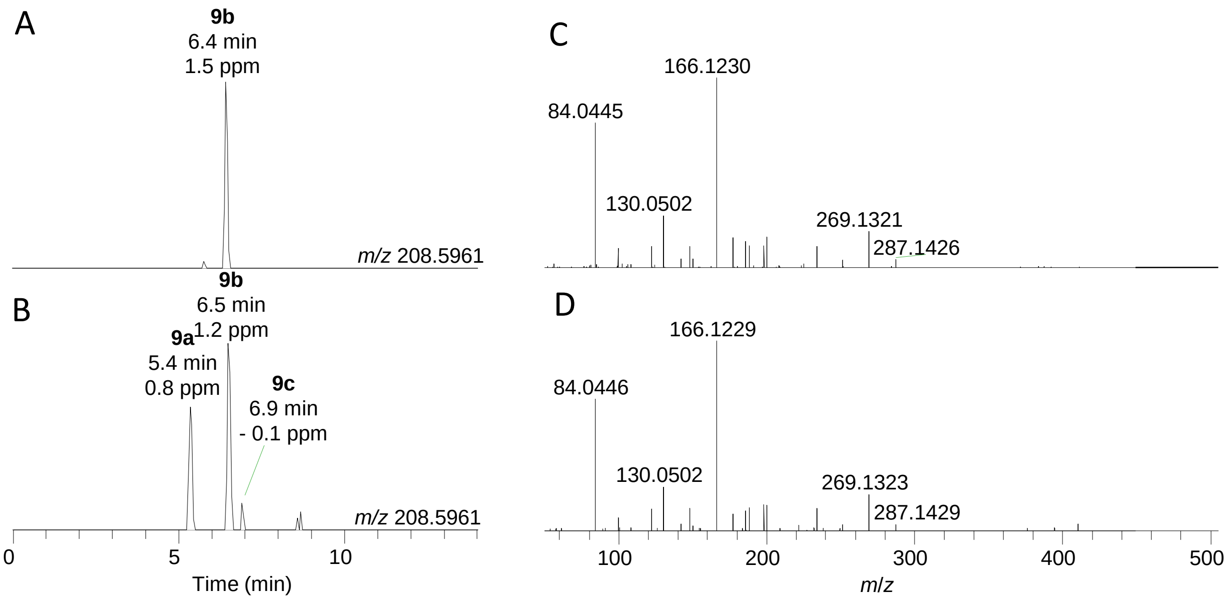

Figure S6: LC–HRMS/MS analysis of γ-Glu-Cys-ATX (**9b**) in a benthic cyanobacterial mat sample (A, C) and isomers **9a**, **9b** and **9c** in a semisynthetic preparation (B, D) showing extracted-ion chromatograms (± 5 ppm) (A, B) and product ion spectra of **9b** collected at a CE = 10 eV (C, D).

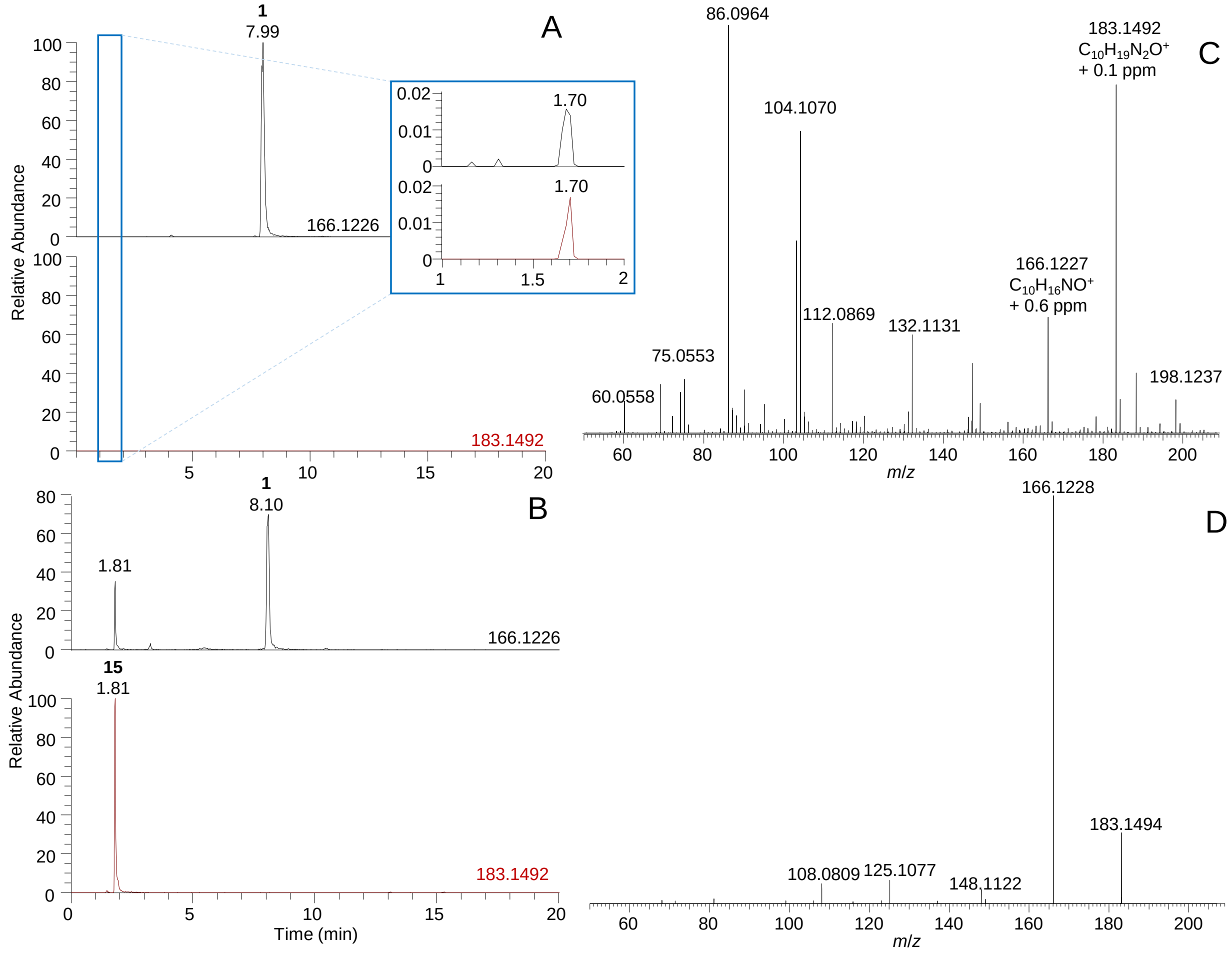

Figure S7: LC–HRMS extracted ion chromatograms (± 3 ppm) for H_2N-ATX (**15**) in a cyanobacterial mat field sample (*m*/*z* 183.1492) extracted in 1:1 MeOH–H_2O without (A) and with 250 mM ammonium bicarbonate (B). Extracted ion chromatograms for ATX (**1**) (*m/z* 166.1662) show the $[M+H–NH_3]^+$ source fragment of **15**. The full scan spectrum of the peak at 1.81 min is shown in pane B (C) and the associated product-ion spectrum of *m*/*z* 183.1492 (D).

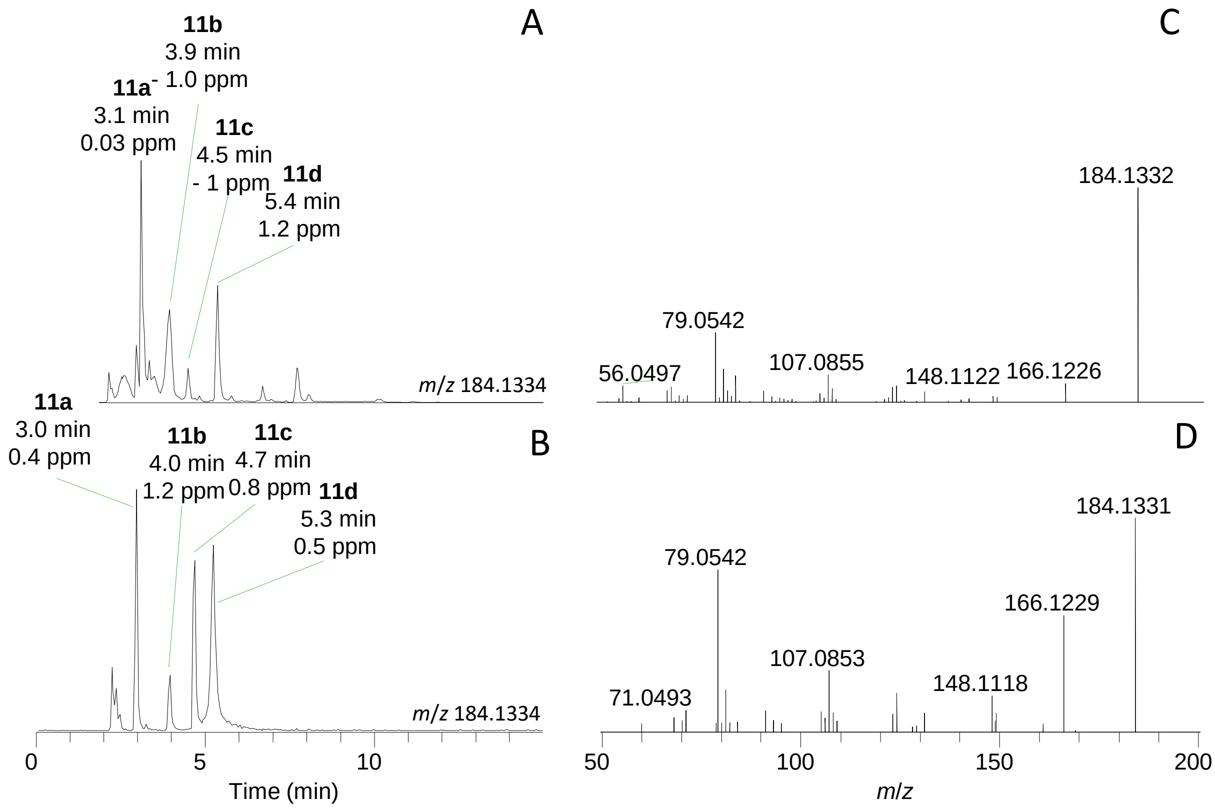

Figure S8: LC–HRMS/MS analysis of isomers of 3-OH-ATX (**11a**, **11b**, **11c** and **11d**) in a benthic cyanobacterial mat sample (A, C) and a semisynthetic preparation (B, D), showing extracted-ion chromatograms (*m*/*z* ± 5 ppm) (A, B) and product-ion spectra of **11b** (C, D).

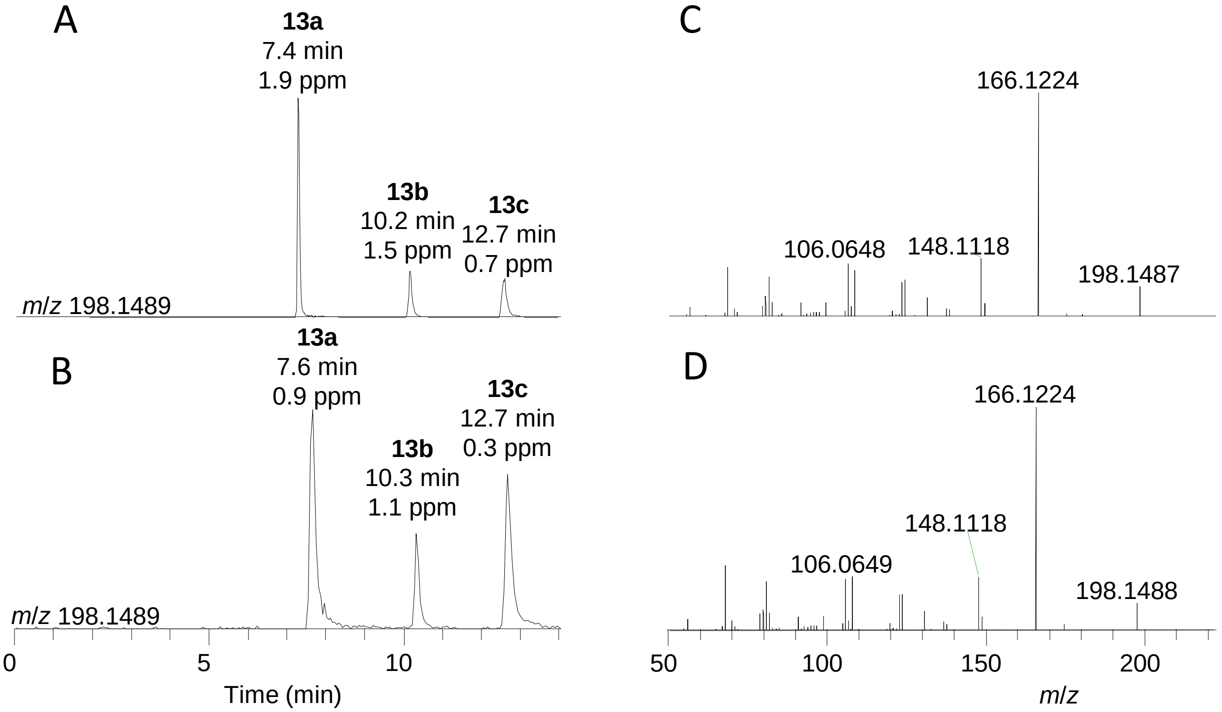

Figure S9: LC–HRMS/MS analysis of isomers of CH_3O-ATX (**13a**, **13b** and **13c**) in a cyanobacterial mat field sample (A, C) and semisynthetic preparation (B, D) showing extracted-ion chromatograms (± 5 ppm) (A, B) and product-ion spectra for **13a**, collected at CE = 10 eV (C, D).

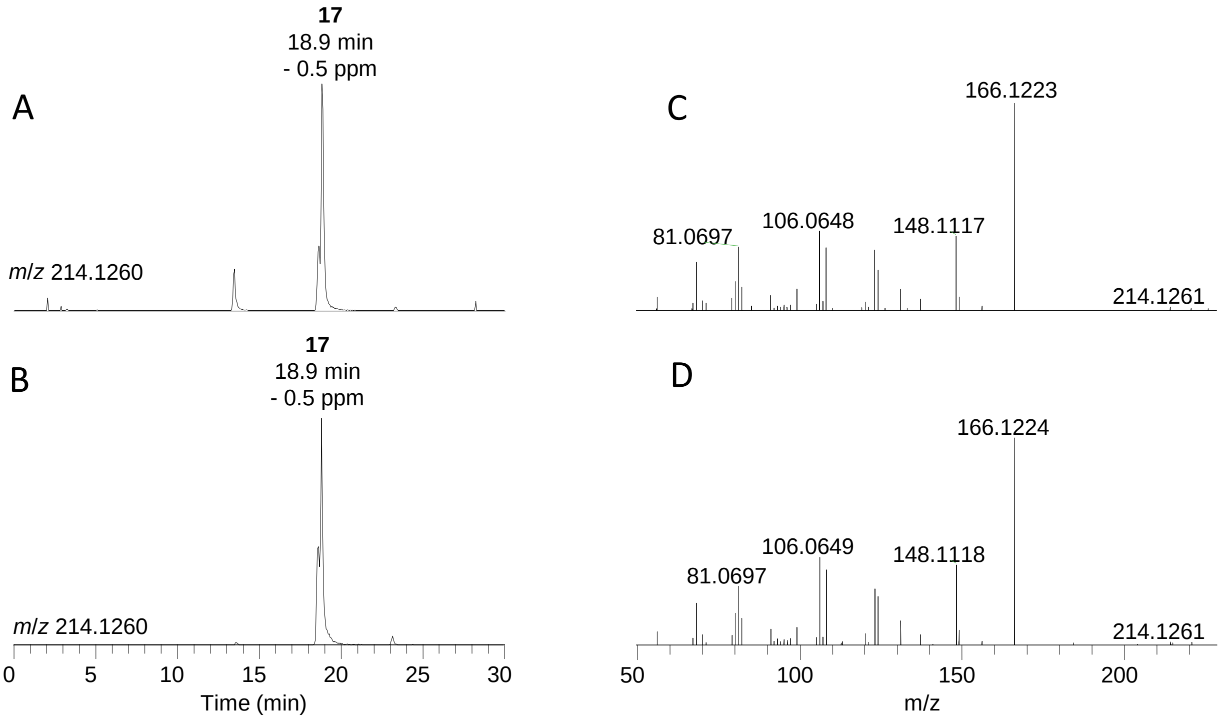

Figure S10: LC–HRMS/MS analysis of CH_3S-ATX (**17**) in a benthic cyanobacterial mat field sample (A, C) and a semisynthetic preparation (B, D), showing extracted-ion chromatograms (± 5 ppm) (A, B) and product-ion spectra of **17** (C, D).

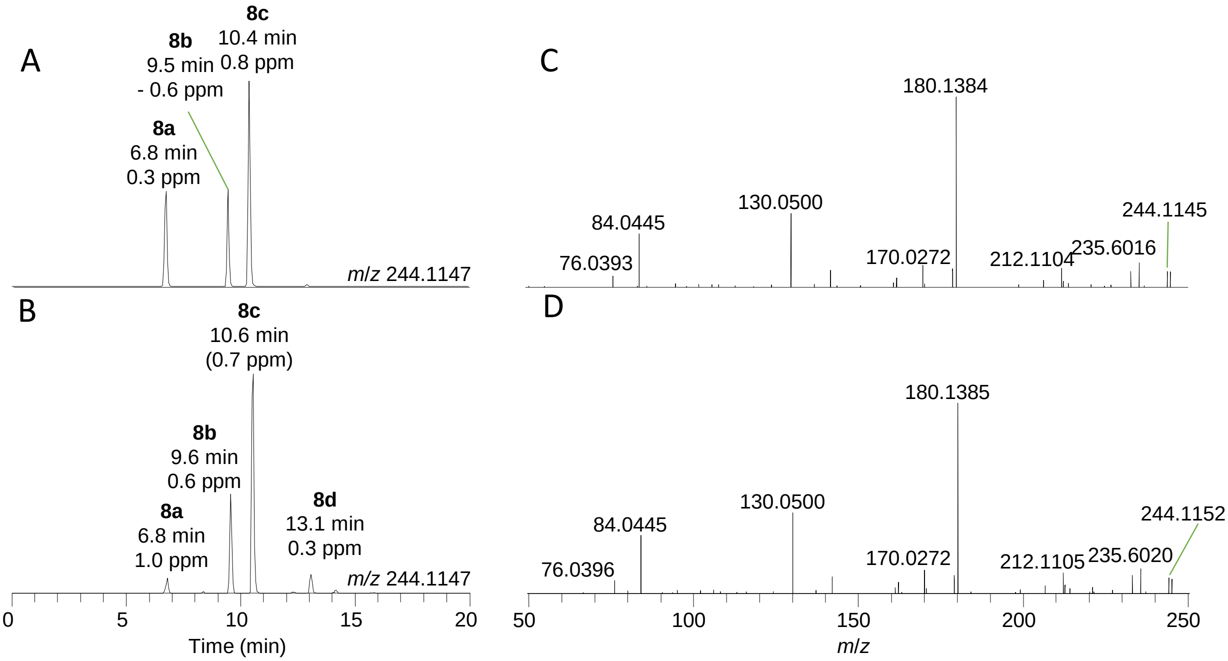

Figure S11: LC–HRMS/MS analysis of isomers of GSH-hATX (**8a**, **8b** and **8c**) in a benthic cyanobacterial mat sample (A, C) and isomers **8a**, **8b**, **8c** and **8d** in a semi-synthetic preparation (B, D), showing extracted-ion chromatogram (*m*/*z* ± 5 ppm) (A, B) and product–ion spectra of **8c**, collected at CE = 10 eV (C, D).

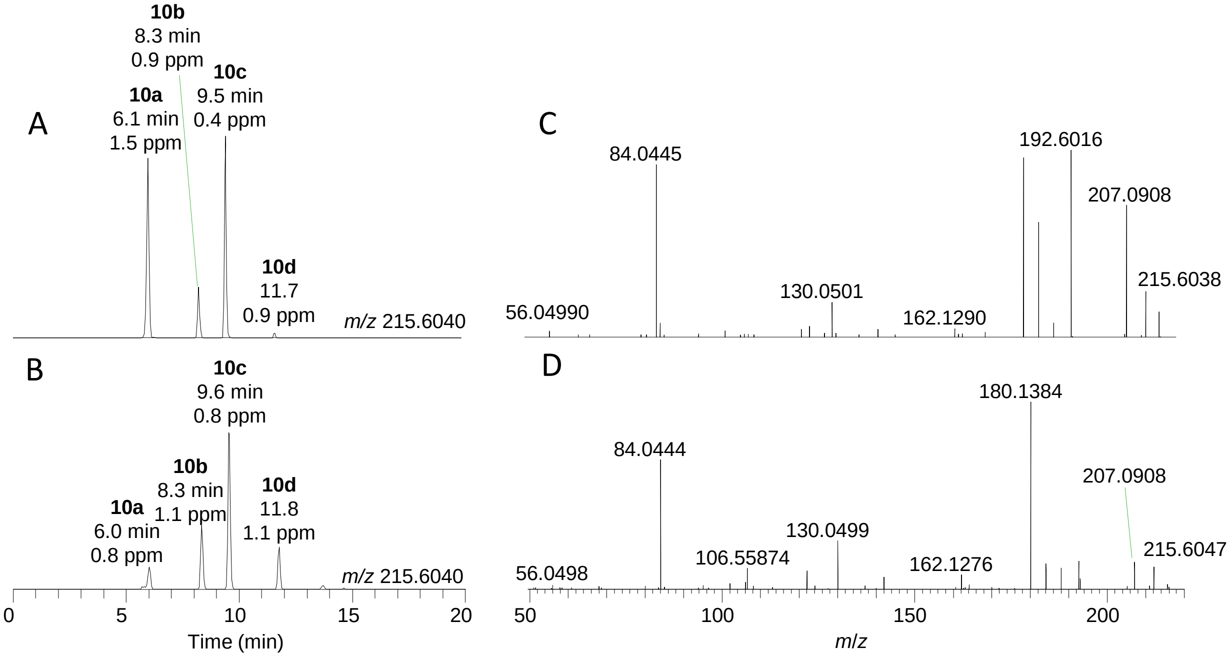

Figure S12: LC–HRMS/MS analysis of isomers of γ-Glu-Cys-hATX (**10a**, **10b**, **10c** and **10d**) in a *K. formosum* culture extract (A, C) and a semisynthetic preparation (B, D), showing extracted-ion chromatograms (± 5 ppm) (A, B) and product-ion spectra (C, D) for **10c** collected at CE = 10 eV.

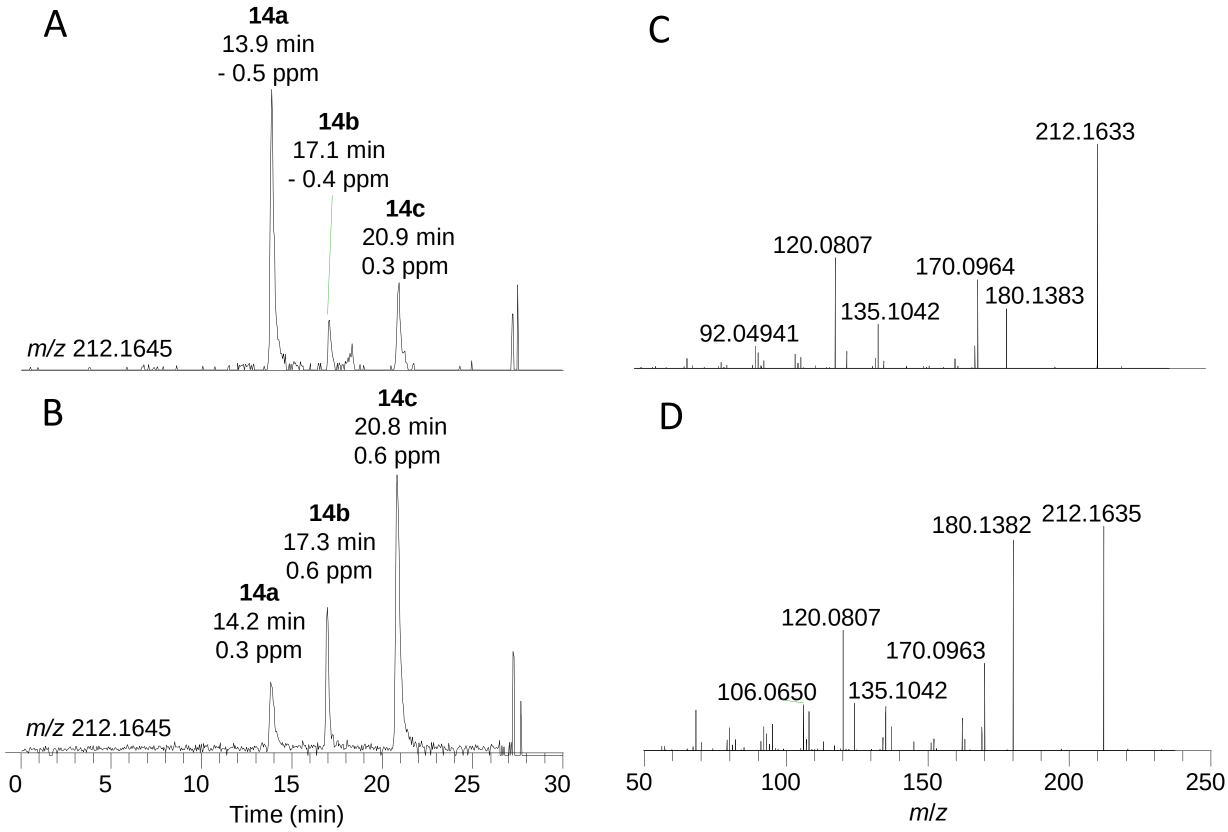

Figure S13: LC–HRMS/MS analysis of isomer of H_3CO-hATX (**14a**, **14b** and **14c**) in an extract of *K. formosum* (A, C) and a semi-synthetic preparation (B, D) showing extracted-ion chromatograms (A, B) and product-ion spectra of **14a** (C, D).

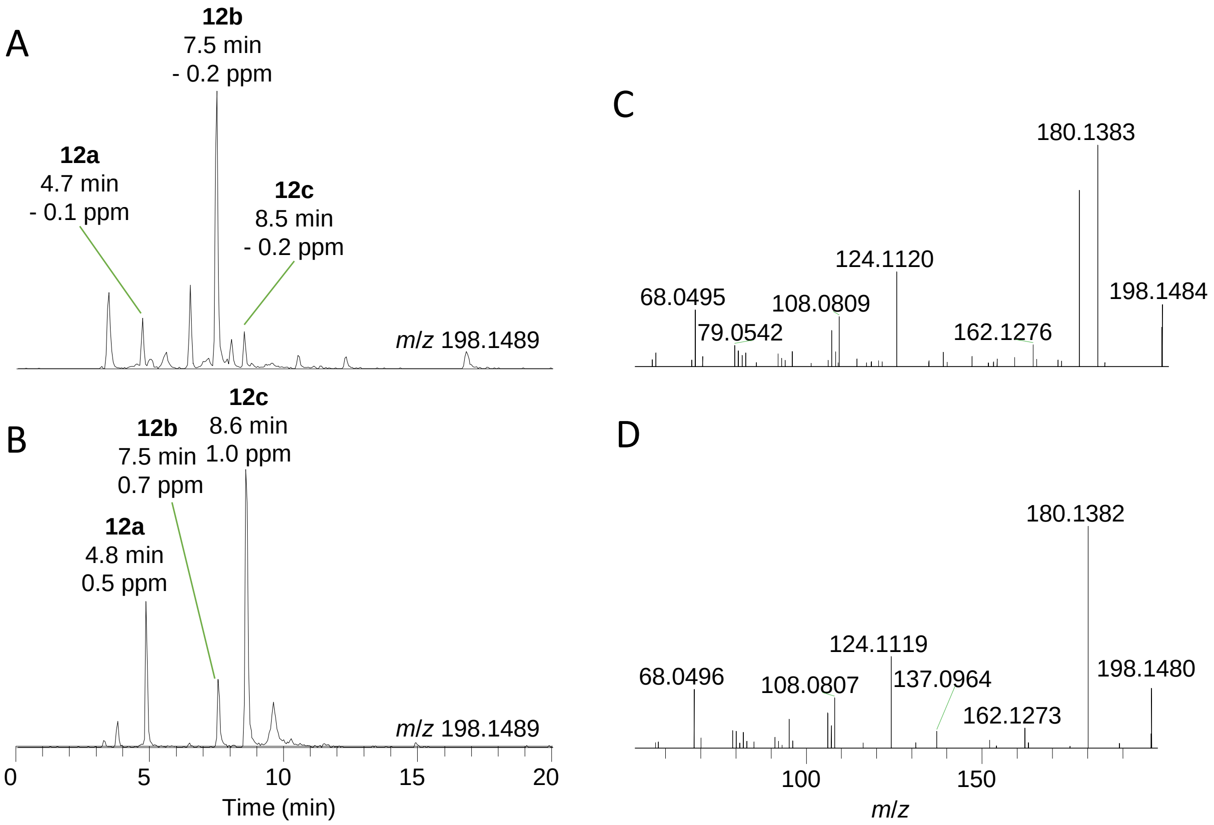

Figure S14: LC–HRMS/MS analysis of isomers of 3-OH-hATX (**12a**, **12b** and **12c**) in a *K. formosum* culture extract (A, C) and a semisynthetic preparation (B, D) showing extracted-ion chromatogram (± 5 ppm) and (A, B) and product-ion spectra for **12b** (C, D).

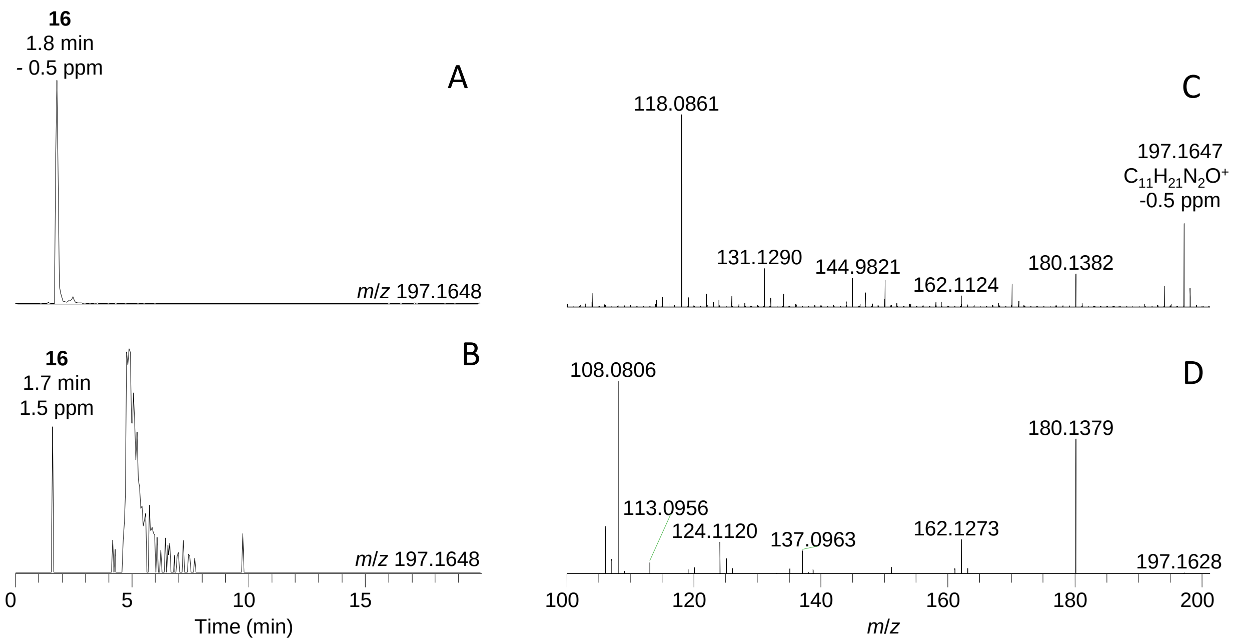

Figure S15: LC–HRMS/MS analysis of H_2N-hATX (**16**) in a *K. formosum* culture including extracted ion chromatograms (*m/z* ± 5 ppm) for samples extracted in 1:1 MeOH:H_2O with (A) and without (B) 250 mM ammonium bicarbonate, as well as full-scan HRMS spectrum of **16** from pane B (C) and MS/MS spectrum of **16** from pane A (D).

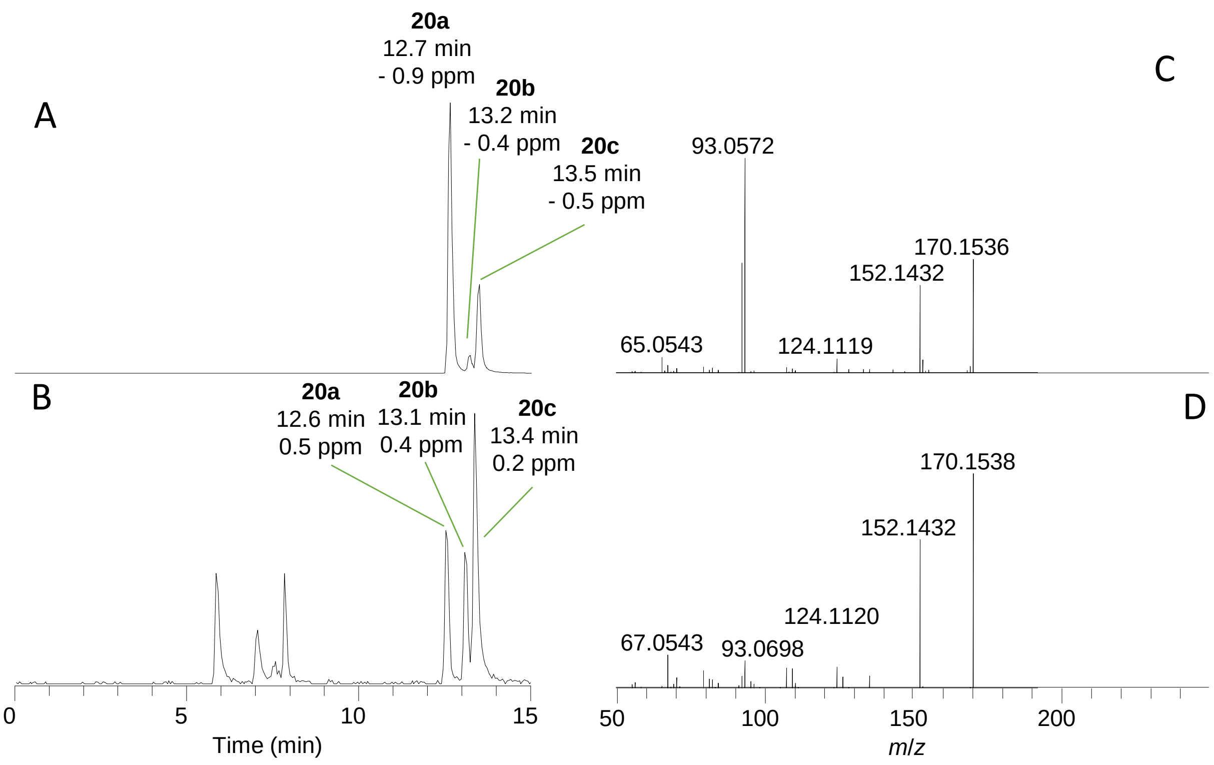

Figure S16: LC–HRMS/MS analysis of isomers of 10-OH-H_2ATX (**20a, 20b** and **20c**) from a benthic cyanobacterial mat sample (A, C) and the reaction between ATX and sodium borohydride (B, D), showing extracted-ion chromatograms (*m*/*z* 170.1539 ± 5 ppm) (A, B) and product-ion spectra (C, D) of **20c** collected at CE = 10 eV.

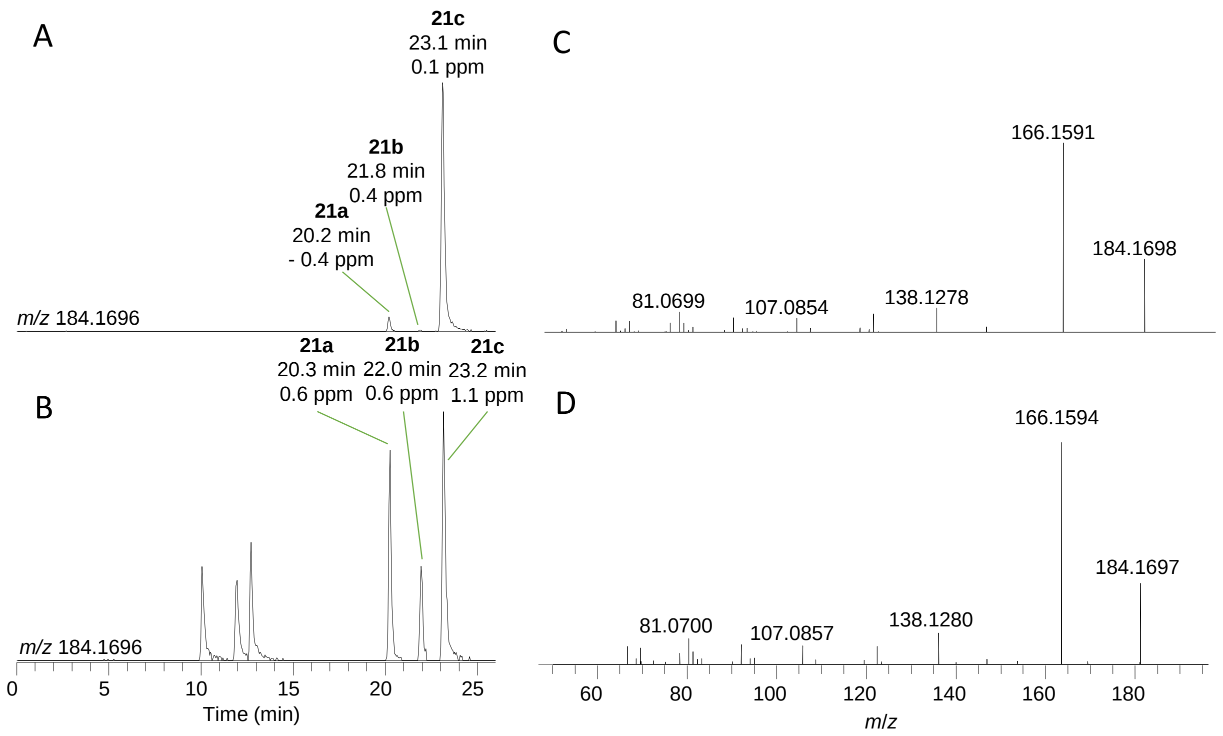

Figure S17: LC–HRMS/MS analysis of isomers of 10-OH-H_2hATX (**21a**, **21b** and **21c**) in an extract of *K. formosum* (A, C) and a reaction between hATX and sodium borohydride (B, D) showing extracted-ion chromatograms (*m*/*z* ± 5 ppm) (A, B) and product-ion spectra (C, D) of **21c**, collected at CE = 20 eV.

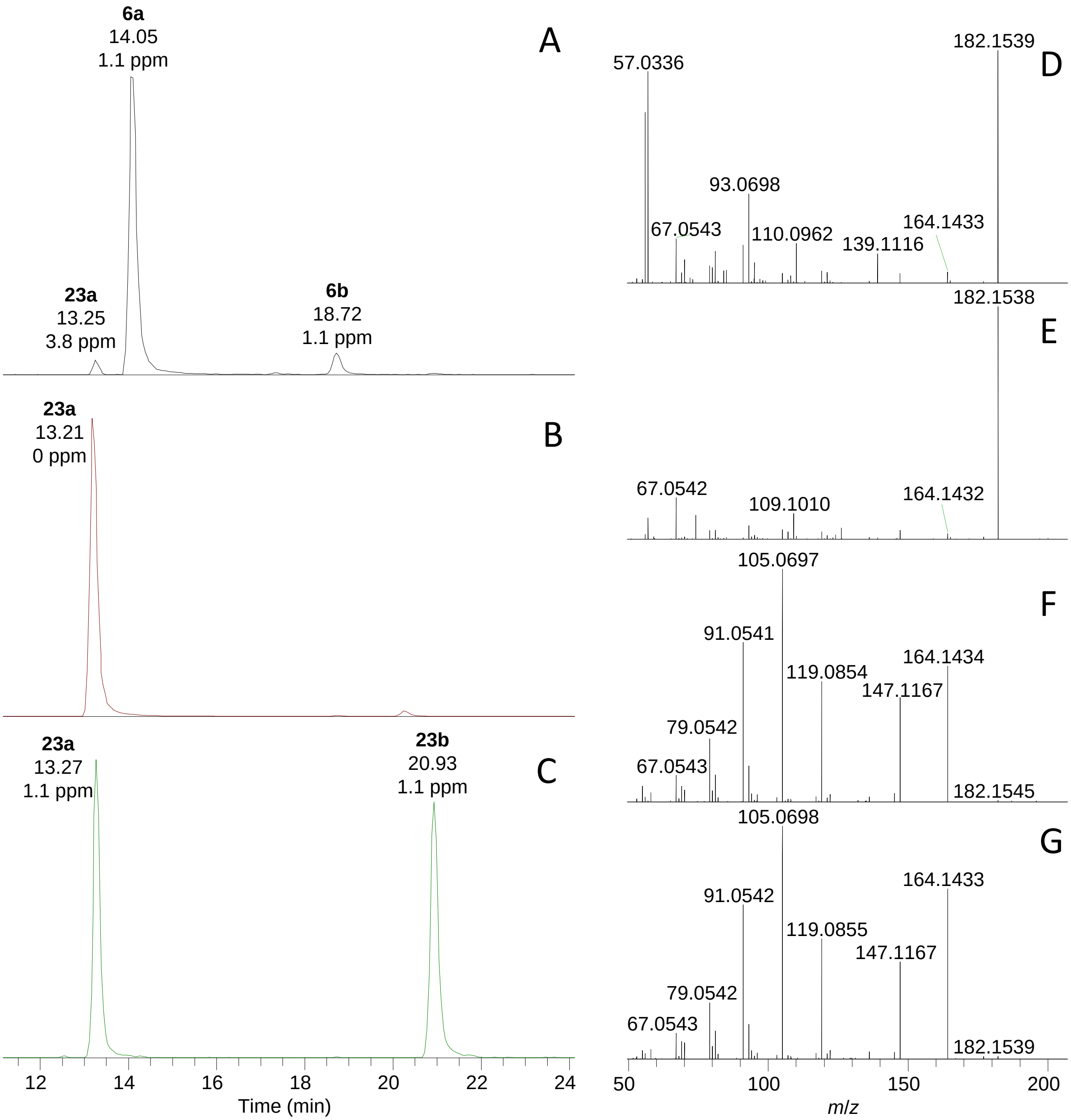

Figure S18: LC–HRMS/MS analysis of *cis*-H_2hATX (**6a**), *trans*-H_2hATX (**6b**) and isomers of 10-OH-hATX (**23a and 23b**) showing extracted-ion chromatograms of *m*/*z* 182.1539 ± 5 ppm in a benthic cyanobacterial mat field sample (A), *K. formosum* culture (B) and the reduction of hATX with $NaBH_4$ (C). Collision-induced dissociation spectra from **6a** (D) and **6b** (E) are from A, **23a** (F) from B and **23b** (G) from C.

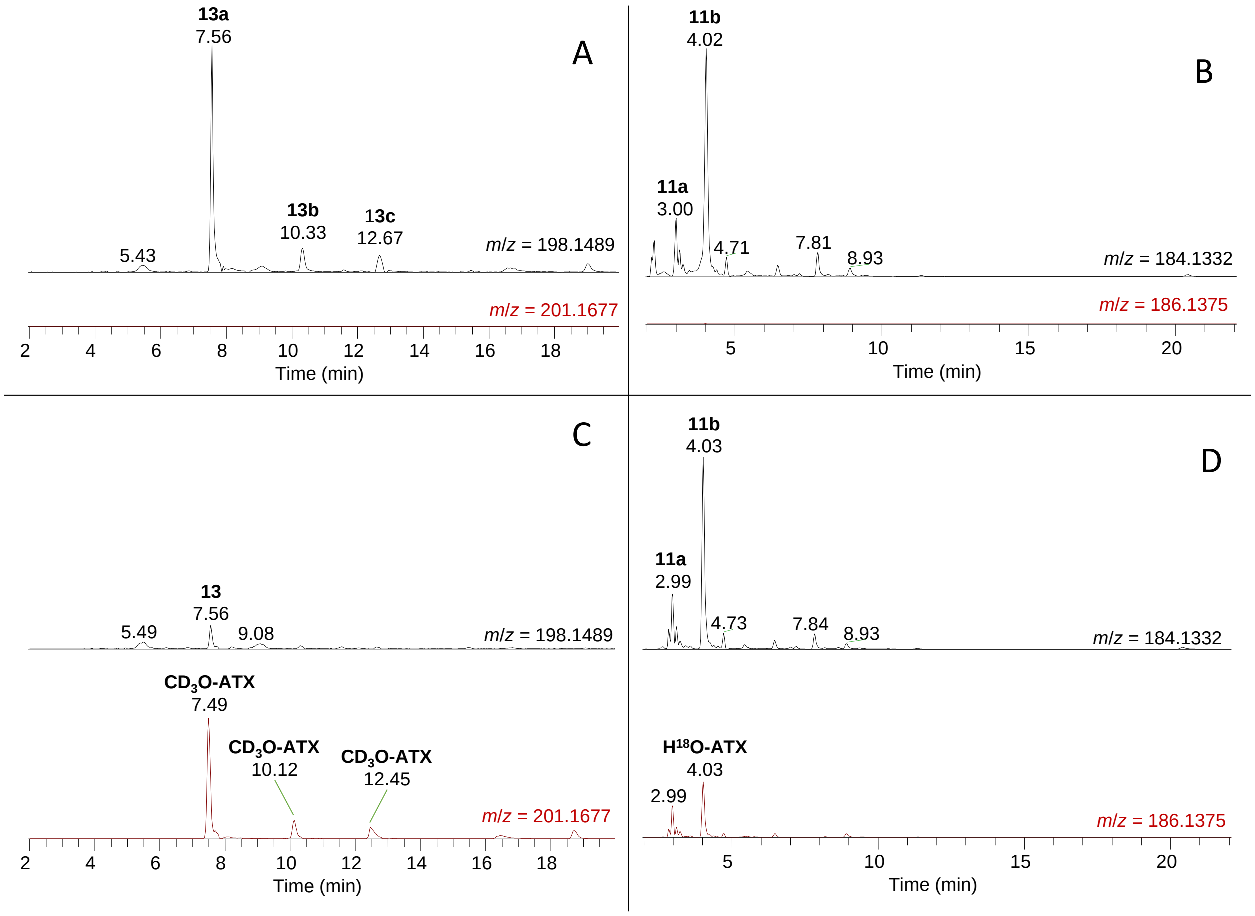

Figure S19: Extracted-ion chromatograms (*m/z* ± 3 ppm) from the LC-HRMS analysis of benthic cyanobacterial mat samples prepared in 1:1 MeOH–H_2O (A, B), 1:1 CD_3OH–H_2O (C), and 1:1 H_2O–$H_2{}^{18}O$ (D), showing detection of isomers of CH_3O-ATX (**13a**, **13b** and **13c**) at *m/z* 196.1489 (A, C) and isomer of 3-OH-ATX (**11a**, **11b** and **11c**) at *m/z* 184.1332 (B, D), along with their isotopically labelled analogues CD_3O-ATX at *m/z* 201.1677 and 3-^{18}OH-ATX at *m/z* 186.1375.

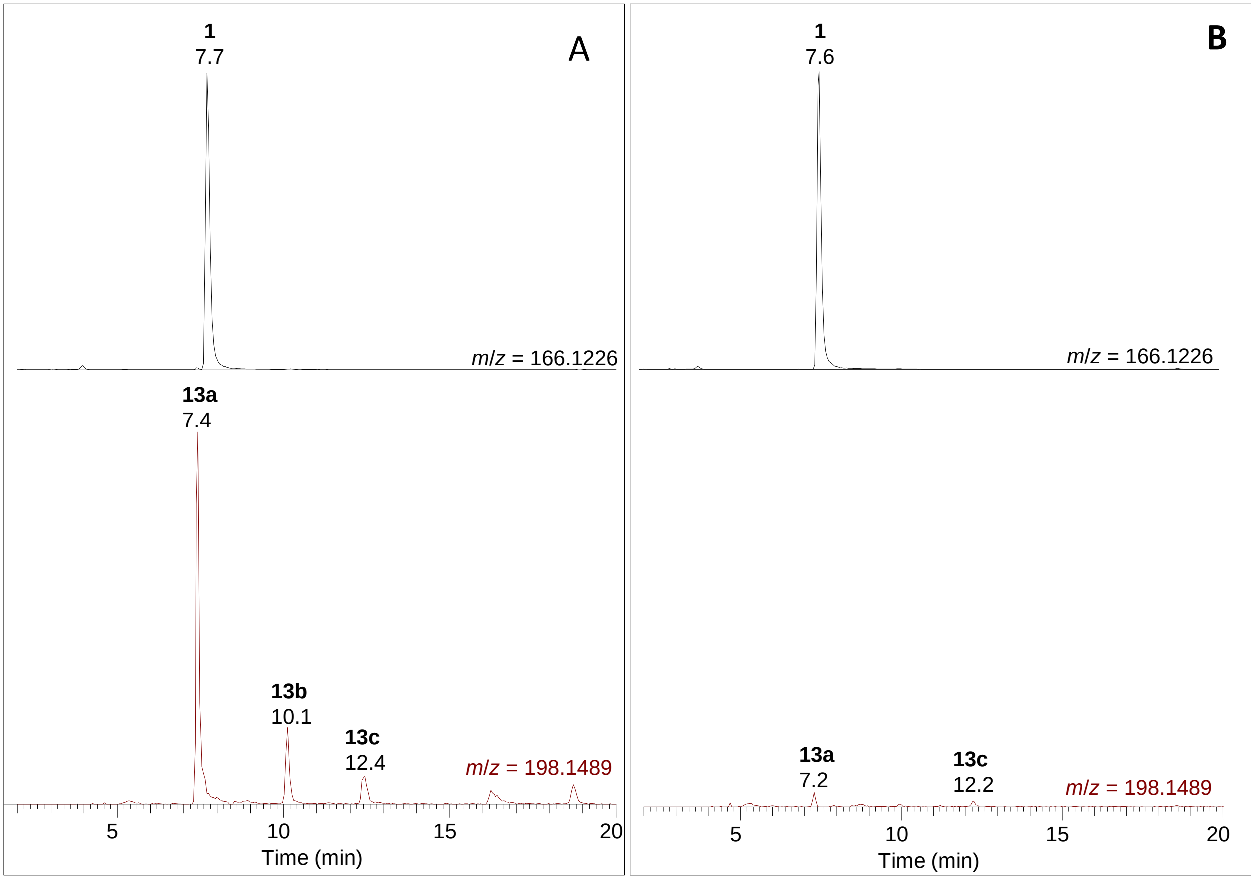

Figure S20: Extracted-ion chromatograms (*m*/*z* ± 5 ppm) from LC–HRMS analysis of preparations of a benthic cyanobacterial mat sample in 1:1 CH_3OH–H_2O (A) and 1:1 CH_3OH–H_2O with 0.1 % formic acid (B) showing ATX (**1**) and isomers of CH_3OH-ATX (**13a**, **13b** and **13c**).

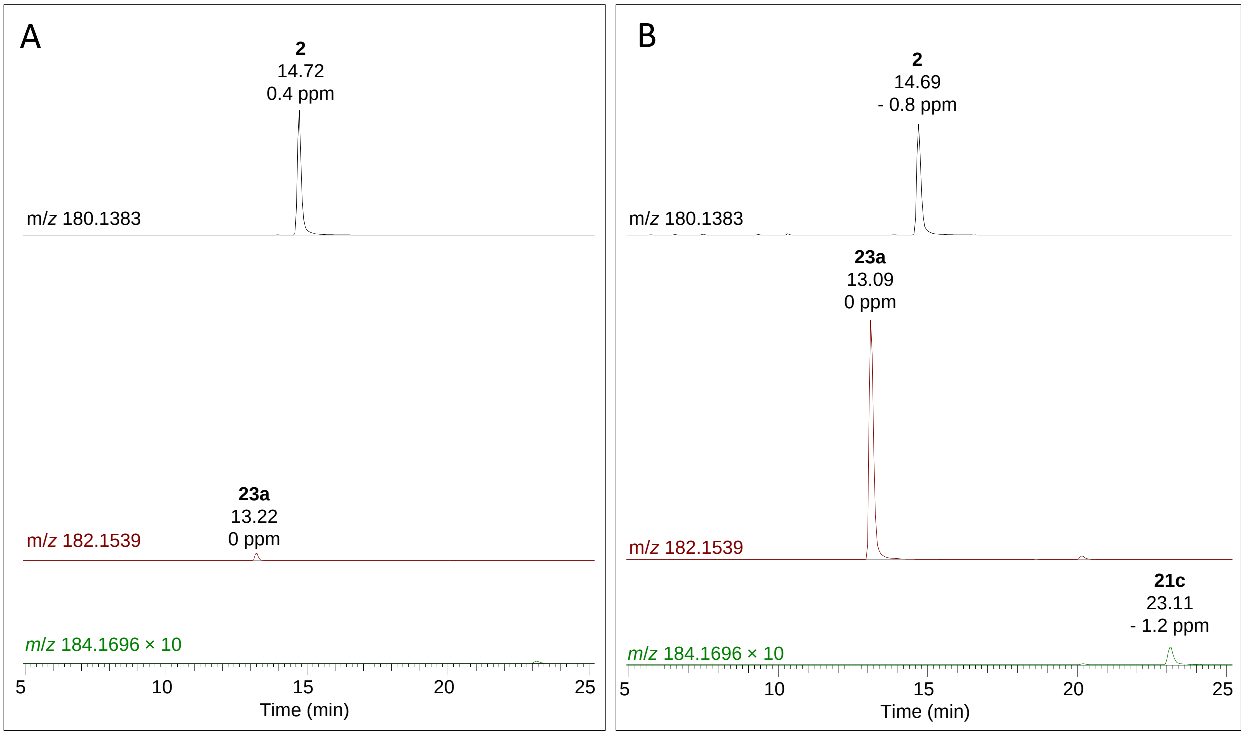

Figure S21: Extracted-ion chromatograms (*m*/*z* ± 5 ppm) from LC–HRMS analysis of preparations of *K. formosum* with MeOH extraction: before (A), or; after (B) freeze–thaw cell lysis, showing hATX (**2**) in both samples, as well as 10-OH-hATX (**23a**) and 10-OH-H_2ATX (**21c**) in the sample extracted after cell lysis (B). All traces are shown to the same scale.

Table S2 – Relative LC-HRMS peak areas of ATXs detected in cyanobacterial samples.

Reference Samples			% Peak Area Relative to Total																					Total %		
Sample Name		Total Targeted ATXs (mg/kg) [a]	ATX	H_2ATX	GSH-ATX	γ-Glu-Cys-ATX	3-OH-ATX	CH_3O-ATX	H_2N-ATX	CH_3SH-ATX	epoxyATX	10-OH-H_2ATX	10-OH-ATX	hATX	H_2hATX	GSH-hATX	γ-Glu-Cys-hATX	3-OH-hATX	CH_3O-hATX	H_2N-hATX	epoxyhATX	10-OH-H_2hATX	10-OH-hATX	Targeted [c]	Conjugates [d]	10-OH-ATXs [e]
RM-BGA		112 [3]	54	3.4	0.1	ND	0.50	4.1	ND	ND	1.0	2.1	ND	29	ND	5.7	0.3	ND	ND	ND	ND	ND	ND	86	11	2.1
Kamptonema formosum (NIVA-CYA 92)		30 [3]	0.1	0.02	0.02	0.003	0.005	ND	0.04	ND	ND	0.01	0.2	26	1.4	10	1.5	0.7	0.2	0.003	0.03	0.6	59	26	14	60
Cuspidothrix issatschenkoi (CAW BG02)		0.12 [3]	56	21	0.8	0.02	0.49	1.2	0.1	ND	2.5	18	ND	ND	ND	ND	ND	ND	ND	ND	ND	ND	ND	77	2.6	18
Environmental Samples																										
Sampling Site [b]	Sampling Date	Total Targeted ATXs (mg/kg) [a]																								
8	22-Jul-18	155 [2]	3.4	44	0.08	0.02	12	0.3	ND	0.002	0.2	37	0.2	0.6	2.2	0.01	0.002	0.05	ND	ND	0.03	0.5	0.01	48	14	38
3	26-Aug-19	60 [4]	39	36	0.03	0.1	1.8	0.5	0.002	0.8	0.2	20	0.7	0.5	0.4	ND	0.002	0.004	ND	ND	0.01	0.08	0.02	76	2.9	20
3	15-Aug-19	60 [5]	59	22	0.3	0.01	1.4	0.1	ND	0.003	0.3	15	0.7	0.4	0.4	0.00	ND	ND	ND	ND	0.01	0.1	0.01	81	2.3	16
6	31-Jul-19	52 [5]	82	7.5	0.01	0.002	1.5	0.2	ND	0.002	0.8	7.1	0.7	0.5	0.1	ND	ND	ND	ND	ND	0.01	0.04	0.007	90	1.8	7.8
4	31-Jul-19	33 [5]	58	14	0.03	0.01	1.4	0.1	ND	0.001	0.6	21	0.7	2.5	0.8	0.00	ND	ND	ND	ND	0.01	0.3	0.04	75	2.3	22
3	30-Jul-19	17 [5]	84	8.3	0.002	ND	1.9	0.1	ND	0.002	0.9	2.6	2.0	0.5	0.1	ND	ND	0.02	ND	ND	0.02	0.04	0.02	92	2.0	4.7
3	30-Jul-19	12 [5]	88	4.2	0.001	0.001	2.1	0.1	ND	0.001	0.7	1.6	2.5	0.5	0.1	ND	ND	ND	ND	ND	0.05	0.03	0.02	93	2.3	4.1
3	18-Jul-19	6.9 [5]	77	12	0.02	0.002	1.0	0.04	ND	ND	0.9	7.0	0.5	1.5	0.8	ND	ND	ND	ND	ND	0.005	0.07	0.01	90	1.9	7.6
3	12-Sep-19	0.76 [5]	41	33	0.02	ND	0.8	0.01	ND	ND	0	25	ND	0.4	0.2	ND	ND	ND	ND	ND	ND	0.02	ND	74	1.0	25
5	30-Jul-19	0.42 [5]	86	8.7	ND	ND	0.9	ND	ND	ND	N/D	2.4	1.5	0.4	0.0	ND	ND	ND	ND	ND	ND	0.02	ND	95	1.0	3.9
3	27-Sep-19	0.32 [5]	39	41	0.4	0.2	2.5	ND	ND	ND	ND	13	ND	0.2	0.4	ND	ND	ND	ND	ND	0.8	0.03	ND	80	3.5	14
7	31-Jul-19	0.26 [5]	35	34	ND	ND	1.1	ND	ND	ND	ND	26	0.9	1.2	1.2	ND	0.3	ND	ND	ND	0.05	0.05	ND	71	2.6	27
4	31-Jul-19	0.18 [5]	32	47	ND	ND	1.1	0.2	ND	ND	ND	19	0.3	0.6	ND	ND	ND	ND	ND	ND	0.06	ND	ND	79	1.3	19
6	31-Jul-19	0.078 [5]	70	17	ND	ND	0.7	0.2	ND	ND	ND	8.7	3.5	0.6	ND	ND	ND	ND	ND	ND	ND	ND	ND	87	0.9	12
1	31-Jul-19	0.014 [5]	39	18	ND	ND	1.7	ND	ND	ND	ND	30	8.4	3.3	ND	ND	ND	ND	ND	ND	ND	ND	ND	60	1.7	38
8	31-Jul-19	0.010 [5]	10	76	ND	ND	ND	ND	ND	ND	ND	14	ND	1.0	ND	ND	ND	ND	ND	ND	ND	ND	ND	86	ND	14
7	31-Jul-19	0.0092 [5]	84	12	ND	ND	ND	ND	ND	ND	ND	4.1	ND	ND	ND	ND	ND	ND	ND	ND	ND	ND	ND	96	ND	4.1
2	30-Jul-19	0.0049 [5]	58	31	ND	ND	ND	11	ND	ND	ND	ND	ND	ND	ND	ND	ND	ND	ND	ND	ND	ND	ND	89	11	ND
2	30-Jul-19	0.0038 [5]	46	39	ND	ND	ND	15	ND	ND	ND	ND	ND	ND	ND	ND	ND	ND	ND	ND	ND	ND	ND	85	15	ND
3	19-Jun-19	0.0037 [5]	58	24	ND	ND	ND	ND	ND	ND	ND	18	ND	ND	ND	ND	ND	ND	ND	ND	ND	ND	ND	82	ND	18
1	31-Jul-19	0.00027 [5]	83	17	ND	ND	ND	ND	ND	ND	ND	ND	ND	ND	ND	ND	ND	ND	ND	ND	ND	ND	ND	100	ND	ND
2	30-Jul-19	<LOD [5]	100	ND	ND	ND	ND	ND	ND	ND	ND	ND	ND	ND	ND	ND	ND	ND	ND	ND	ND	ND	ND	100	ND	ND

[a] Sum of ATX, hATX and H_2ATX concentration determined by LC-HRMS as part of previous study (referenced in square brackets). Results given as wet weight with the exception of RM-BGA, which is a freeze-dried sample.

[b] Sampling sites on the Wolastoq near Fredericton, NB, Canada, corresponded to the following coordinates. site 1 (45.958750, -66.827658), site 2 (45.992082, -66.819793), site 3 (45.974617, -66.759316), site 4 (45.967481, -66.745481), site 5 (45.973053, -66.698757), site 6 (45.964856, -66.662421), site 7 (45.973053, -66.698757), site 8 (45.961651, -66.627210)

[c] Sum of peak areas of ATX, hATX and H_2ATX normalized to total peak area of all detected ATXs

[d] Sum of peak areas of GSH, γ-Glu-Cys, H_2O, CH_3OH and CH_3SH conjugates of ATX and hATX normalized to total peak area of all detected ATXs

[e] Sum of peak areas of 10-OH-ATX, 10-OH-H_2ATX, 10-OH-hATX and 10-OH-H_2hATX normalized to total peak area of all detected ATXs

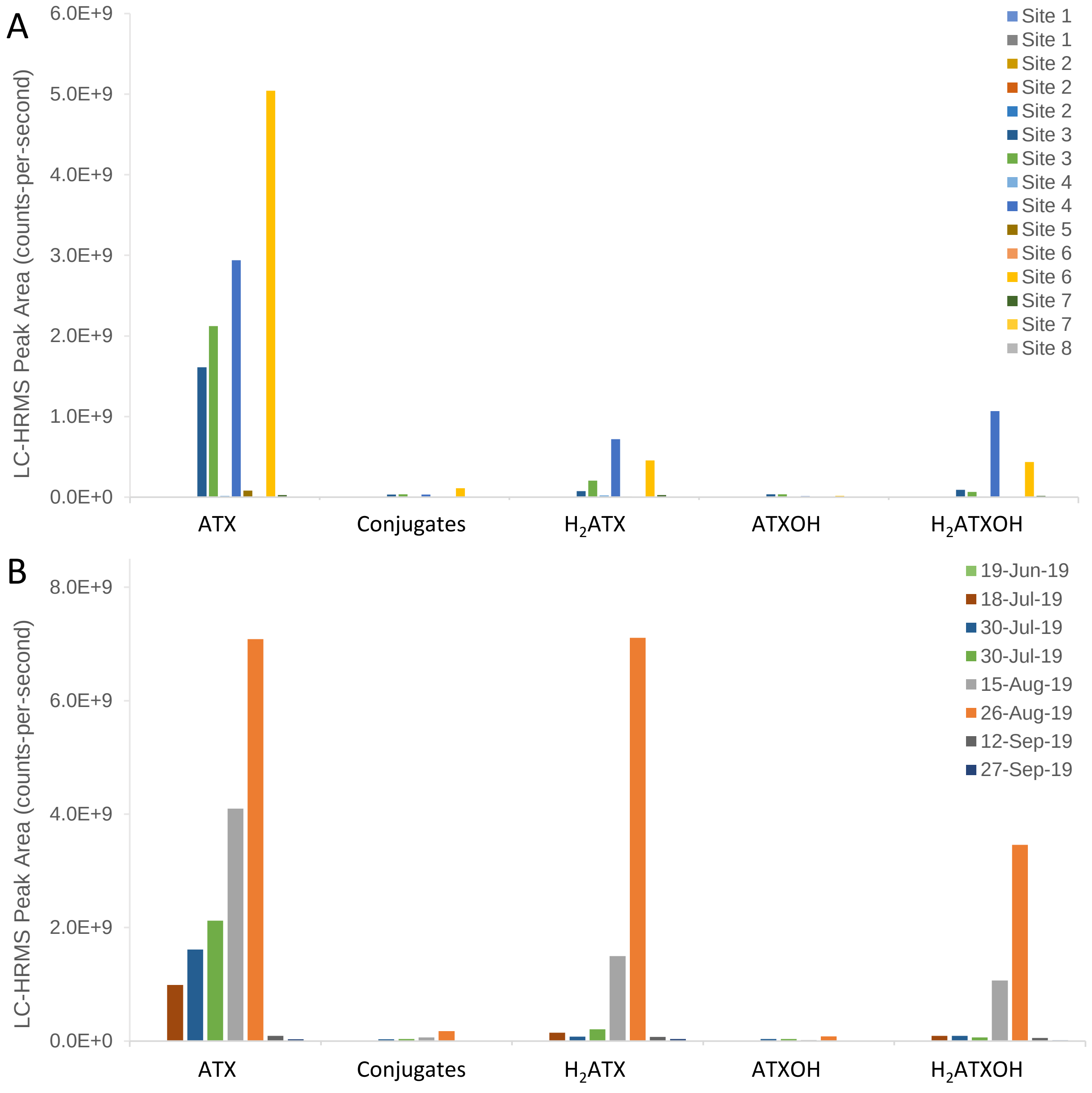

Figure S22: Anatoxin profiles in benthic cyanobacterial mats collected from the Wolastoq near Fredericton, NB, Canada, in Summer 2019 showing spatial variability in samples collected from various sites on July 30th and 31st (A) and temporal trends in samples collected from site 3 throughout the summer (B). The Conjugates grouping represents the sum of all conjugates detected. Sampling sites corresponded to the coordinates specified in Table S2.

References

1. Mejean A, Dalle K, Paci G, Bouchonnet S, Mann S, Pichon V, Ploux O. Dihydroanatoxin-a is biosynthesized from proline in *Cylindrospermum stagnale* PCC 7417: Isotopic incorporation experiments and mass spectrometry analysis. J Nat Prod. 2016;79:1775-1782. doi:10.1021/acs.jnatprod.6b00189.

2. McCarron P, Rafuse C, Scott S, Lawrence J, Bruce MR, Douthwright E, Murphy C, Reith M, Beach DG. Anatoxins from benthic cyanobacteria responsible for dog mortalities in New Brunswick, Canada. Toxicon. 2023;107086:1-9. doi:10.1016/j.toxicon.2023.107086.

3. Beach DG, Rafuse C, Melanson JE, McCarron P. Rapid quantitative screening of cyanobacteria for production of anatoxins using direct analysis in real time high-resolution mass spectrometry. Rapid Commun Mass Spectrom. 2021;35:e8940. doi:10.1002/rcm.8940.

4. Beach DG, Lawrence J, Bruce M, Stillwell J, Rafuse C, McCarron P (2022) Anatoxins in cyanobacterial mat field samples from Atlantic Canada by direct analysis in real time–high resolution tandem mass spectrometry In: Band-Schmidt CJ (ed) 19th International Conference on Harmful Algae, La Paz, Mexico, 2021. International Society for the Study of Harmful Algal Blooms, pp 151-156. doi:10.5281/zenodo.7035072

5. Beach DG, Bruce M, Lawrence J, McCarron P. Rapid quantitation of anatoxins in benthic cyanobacterial mats using direct analysis in real-time–high-resolution tandem mass spectrometry. Environ Sci Technol. 2022;56:13837-13844. doi:10.1021/acs.est.2c05426.
